# Supplementary material for: Integrating evolutionary and regulatory information with a multispecies approach implicates genes and pathways in obsessive-compulsive disorder
Source: Nat Commun. 2017 Oct 17;8:774. doi: 10.1038/s41467-017-00831-x (PMC5645406; doi:10.1038/s41467-017-00831-x)
Supplement: Supplementary file 1 — Supplementary Information [file 41467_2017_831_MOESM1_ESM.pdf]

SUPPLEMENTARY FIGURES

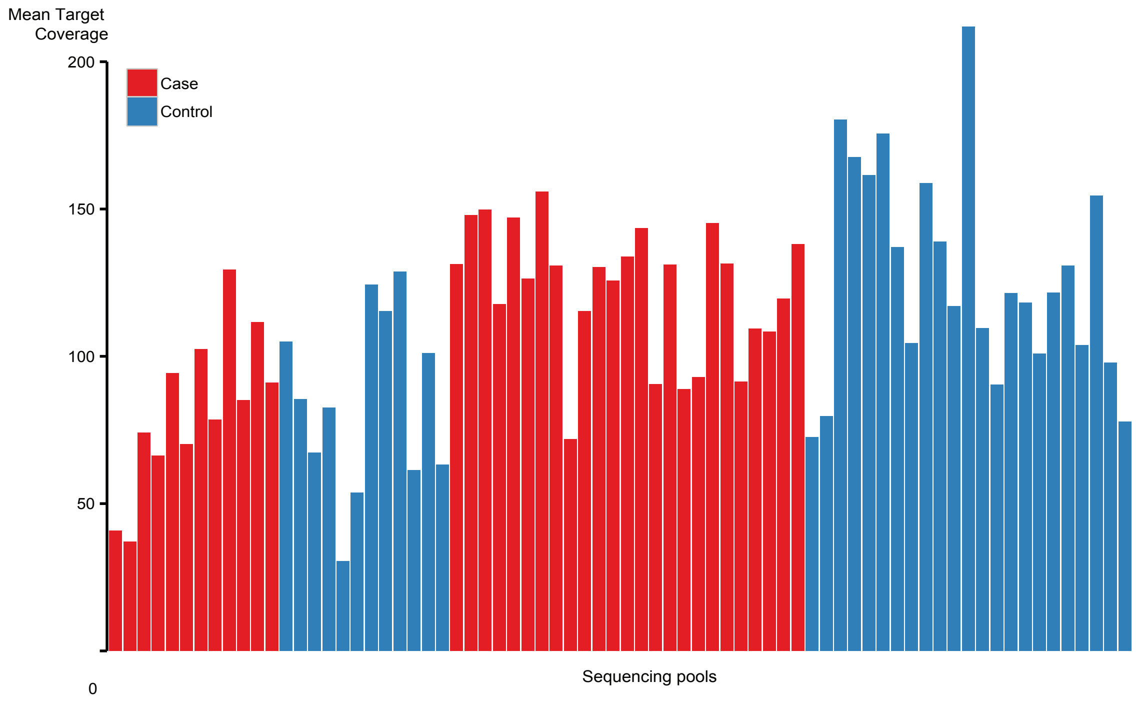

Supplementary Figure 1. Mean target read depth coverage per pool

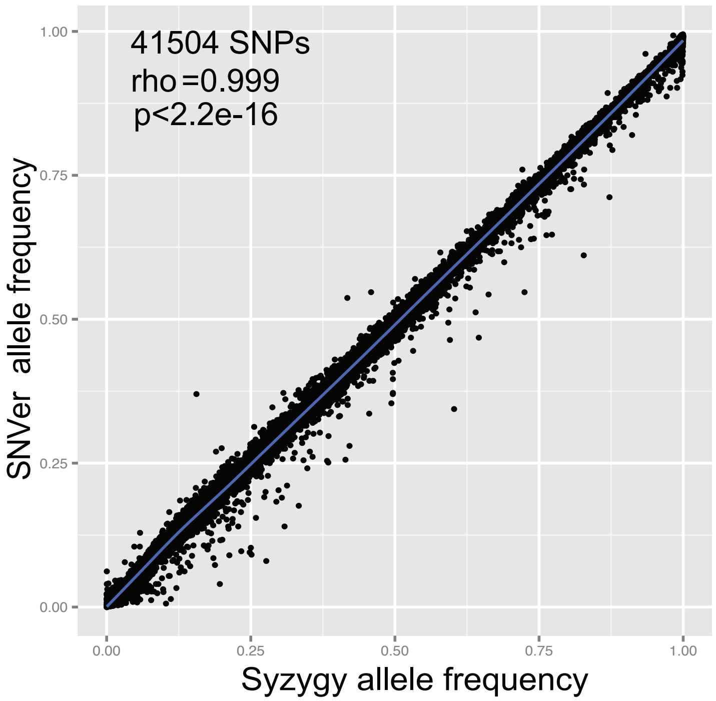

Supplementary Figure 2. Comparison of allele frequencies estimated by Syzygy (x-axis) and SNVer (y-axis) from discovery pooled sequencing. Two-sided Pearson's correlation test confirms almost perfect linear relationship.

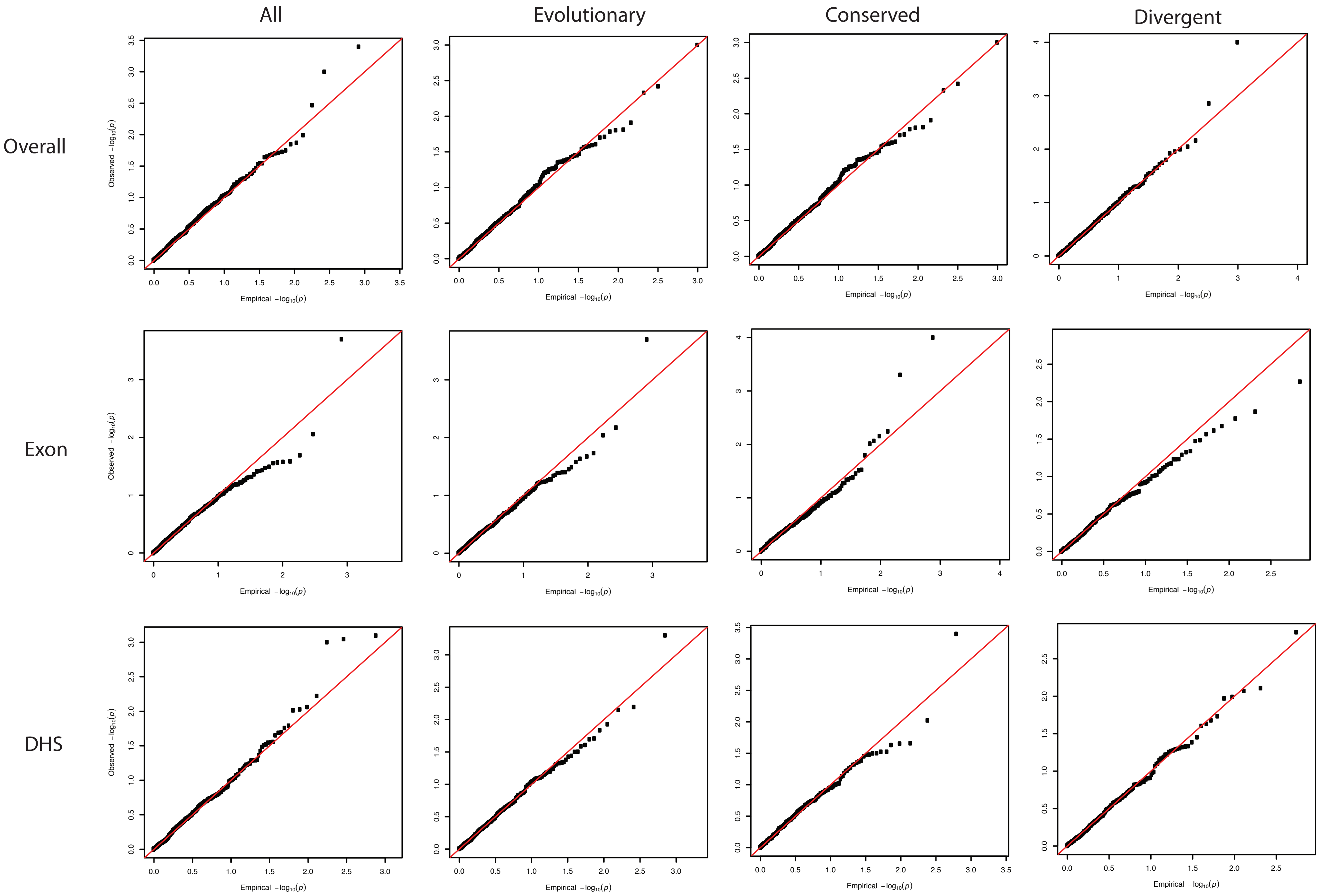

Supplementary Figure 3b. Quantile-quantile plots for individual PolyStrat gene tests corresponding to Figure 1b. Y-axis indicates observed p-values. X-axis indicates empirical expected p-values.

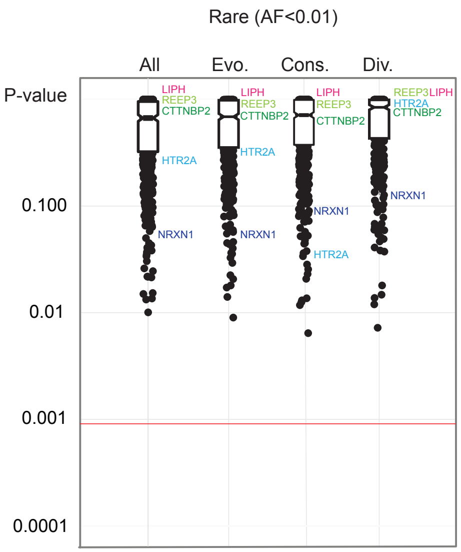

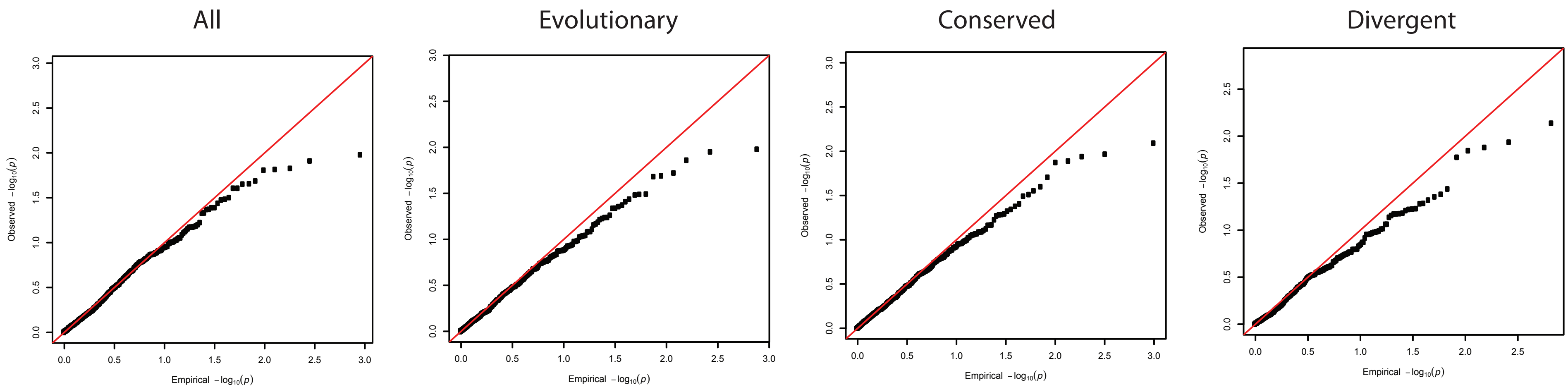

Supplementary Figure 4. Quantile-quantile plots for PolyStrat with rare variant (allele frequency<0.01) filter

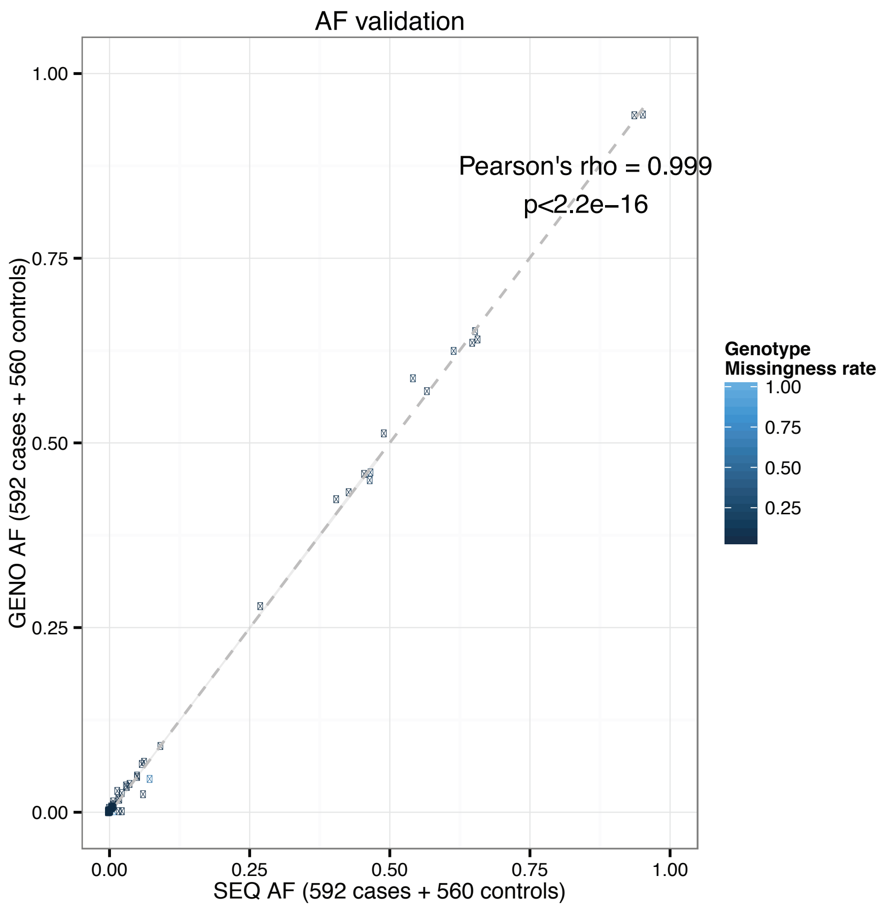

Supplementary Figure 5. Comparison of allele frequencies estimated from pooled sequencing (x-axis, called by Syzygy) and those measured from Sequenom genotyping (y-axis) in discovery sample cohort. Two-sided Pearson's correlation test confirms almost perfect linear relationship.

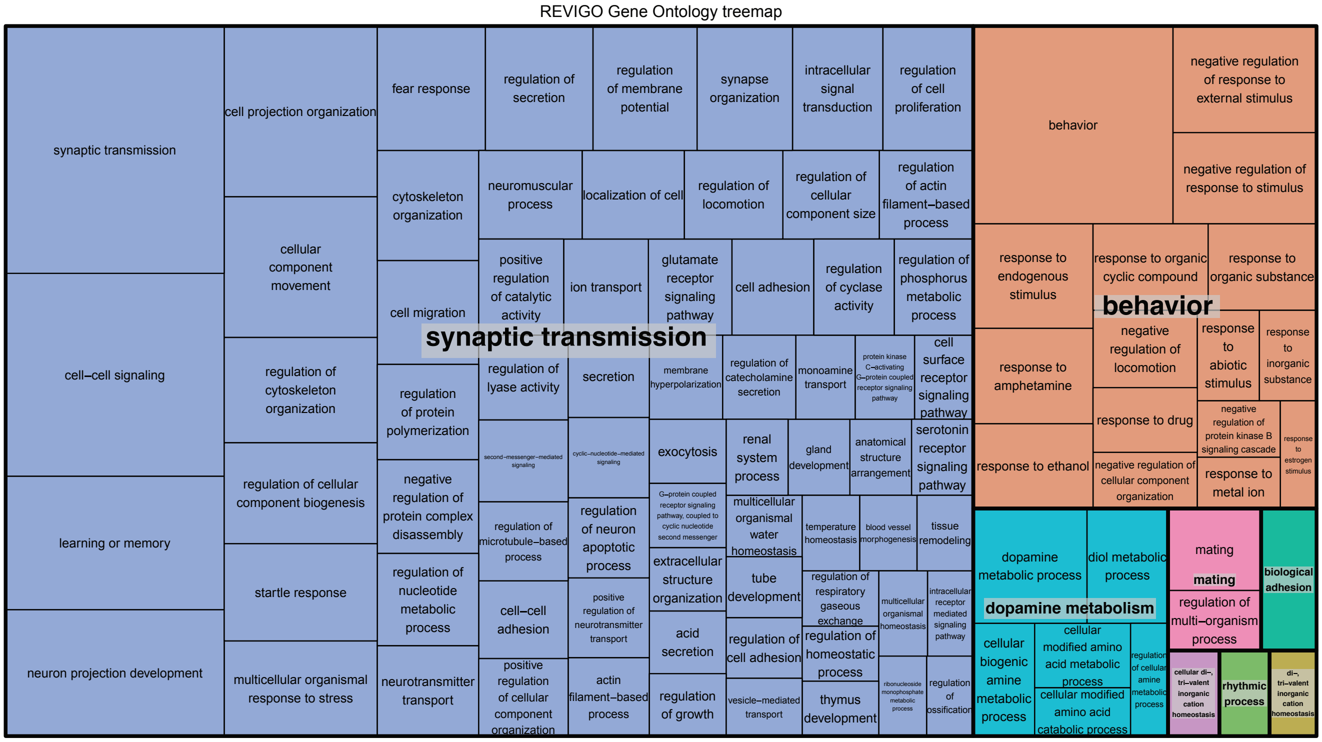

Supplementary Figure 6a. Biological Processes of the 989 GO terms representing 608 sequenced genes

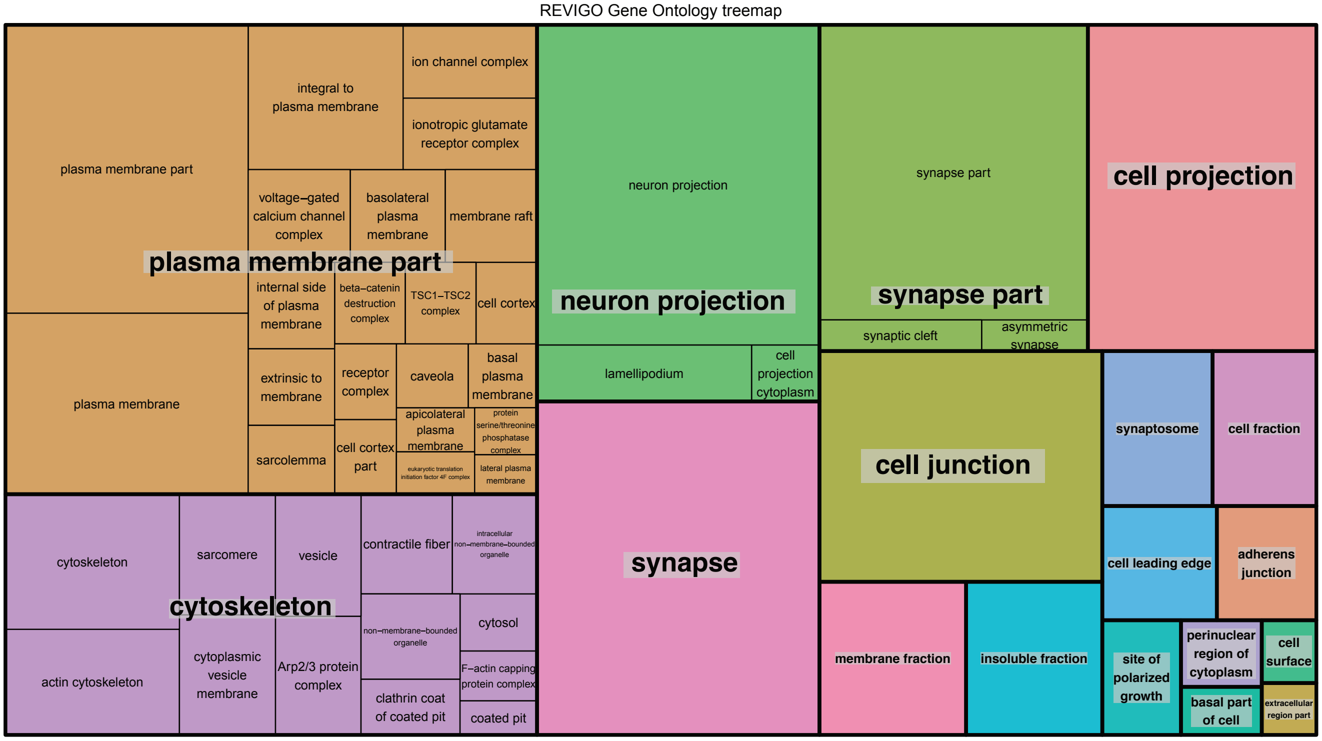

Supplementary Figure 6b. Cellular Components of the 989 GO terms representing 608 sequenced genes

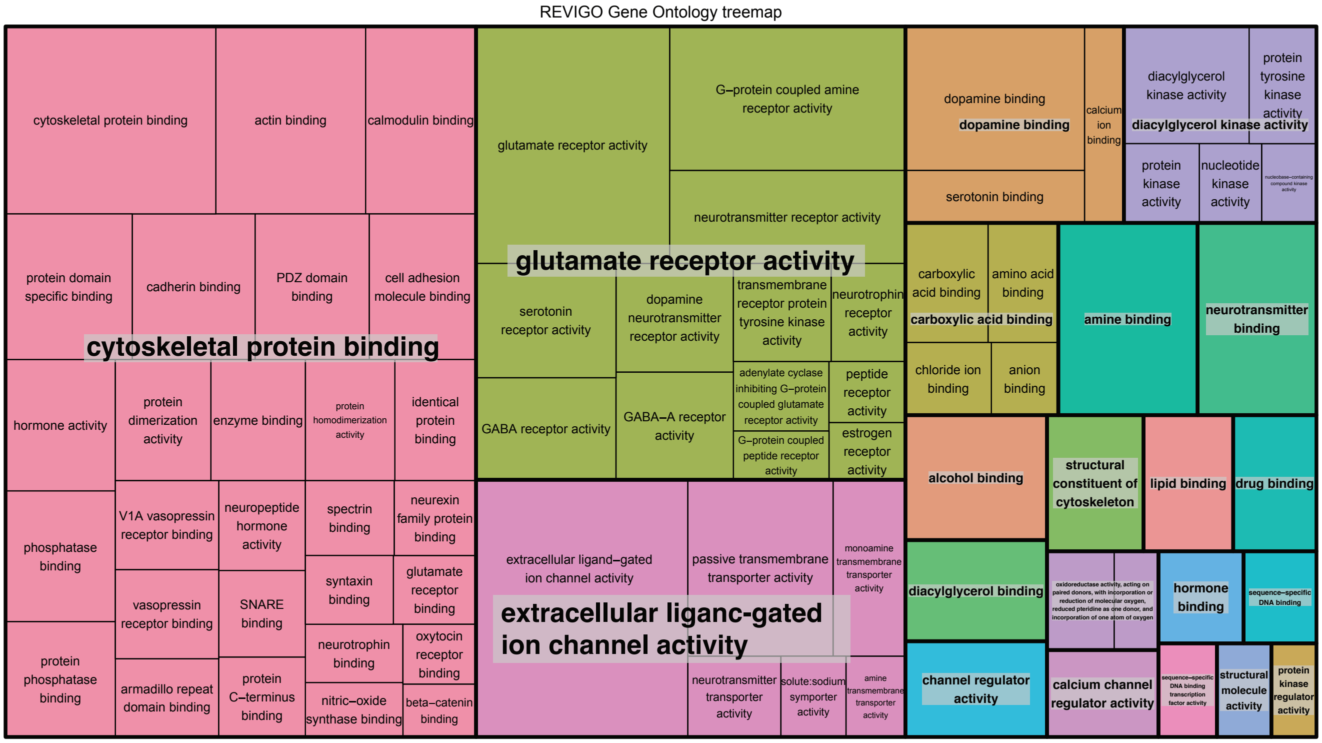

Supplementary Figure 6c. Molecular Functions of the 989 GO terms representing 608 sequenced genes

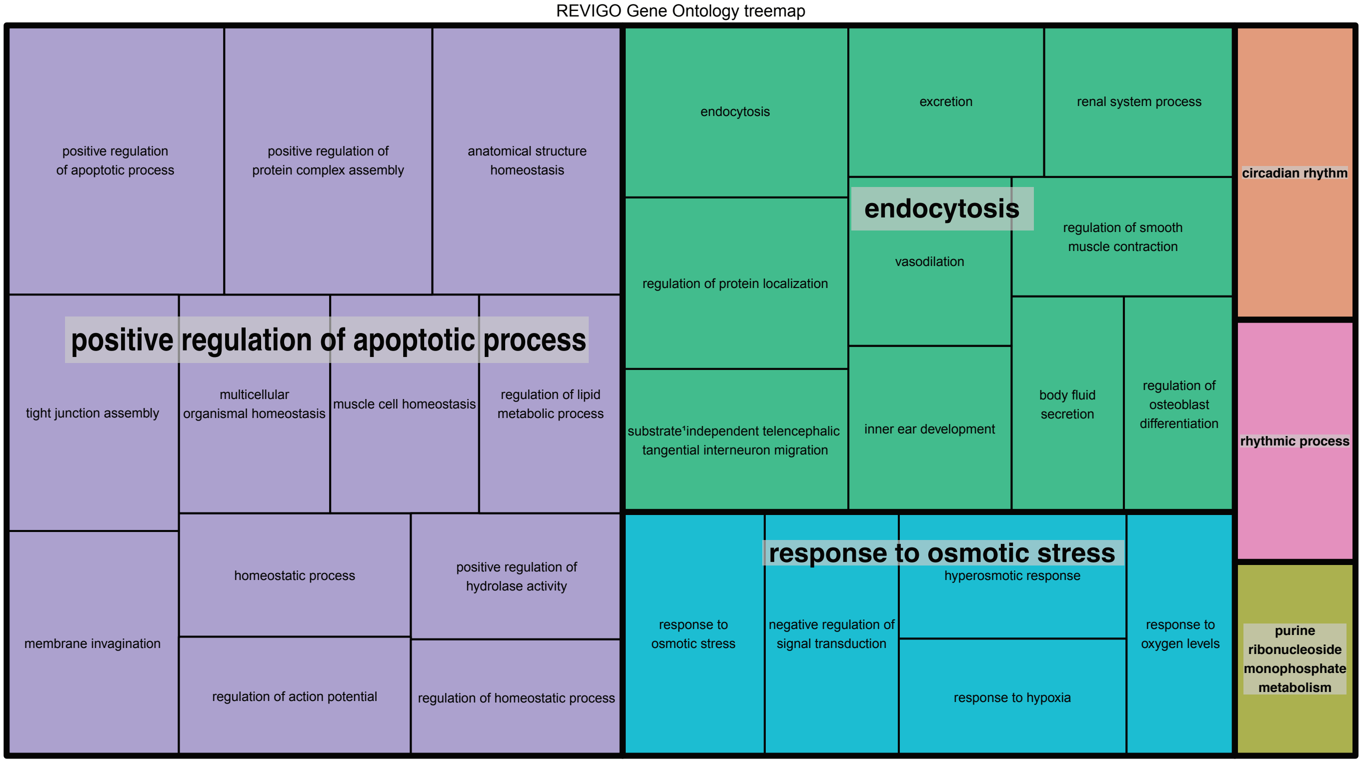

Supplementary Figure 6d. Biological Processes of 82 GO terms with nominal burden (uncorr. p<0.05) of non-reference alleles in cases

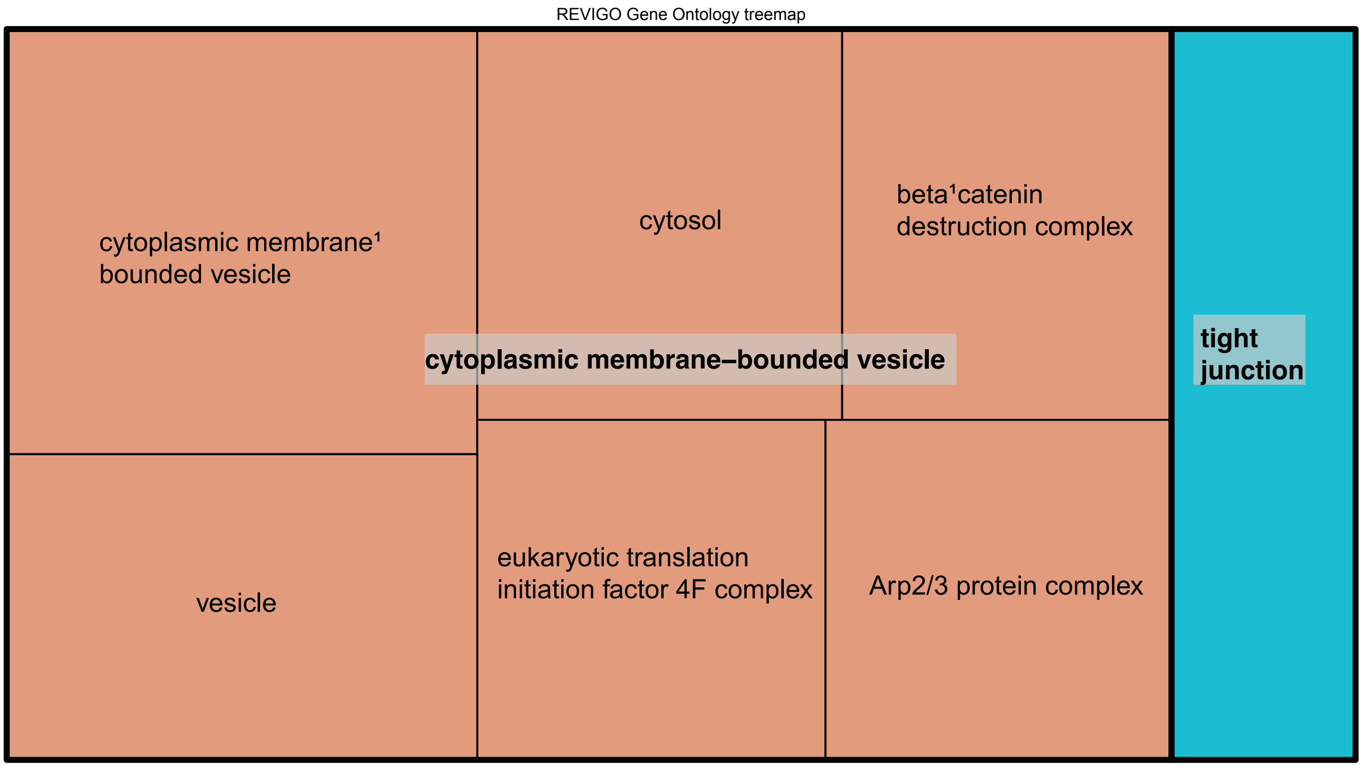

Supplementary Figure 6e. Cellular Component of 82 GO terms with nominal burden (uncorr. $p<0.05$) of non-reference alleles in cases

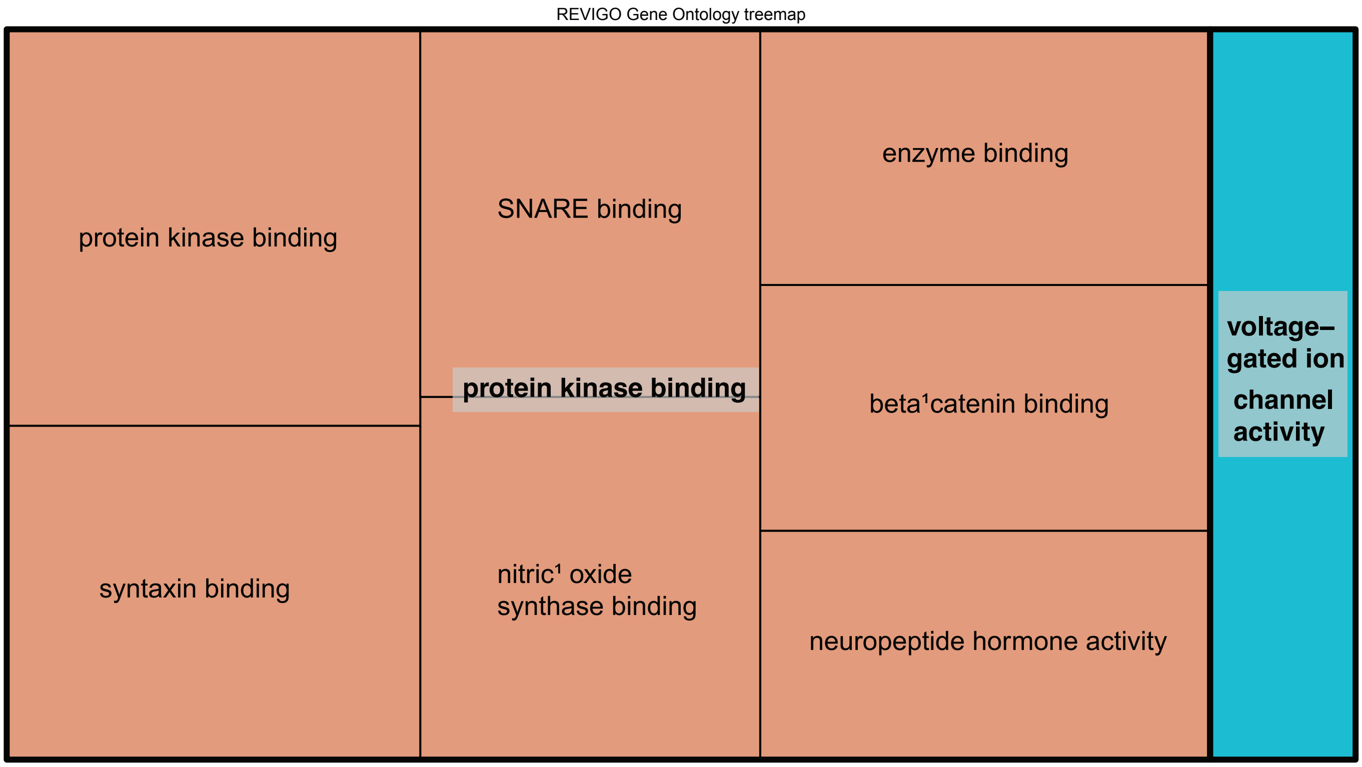

Supplementary Figure 6f. Molecular Function of 82 GO terms with nominal burden (uncorr. $p<0.05$) of non-reference alleles in cases

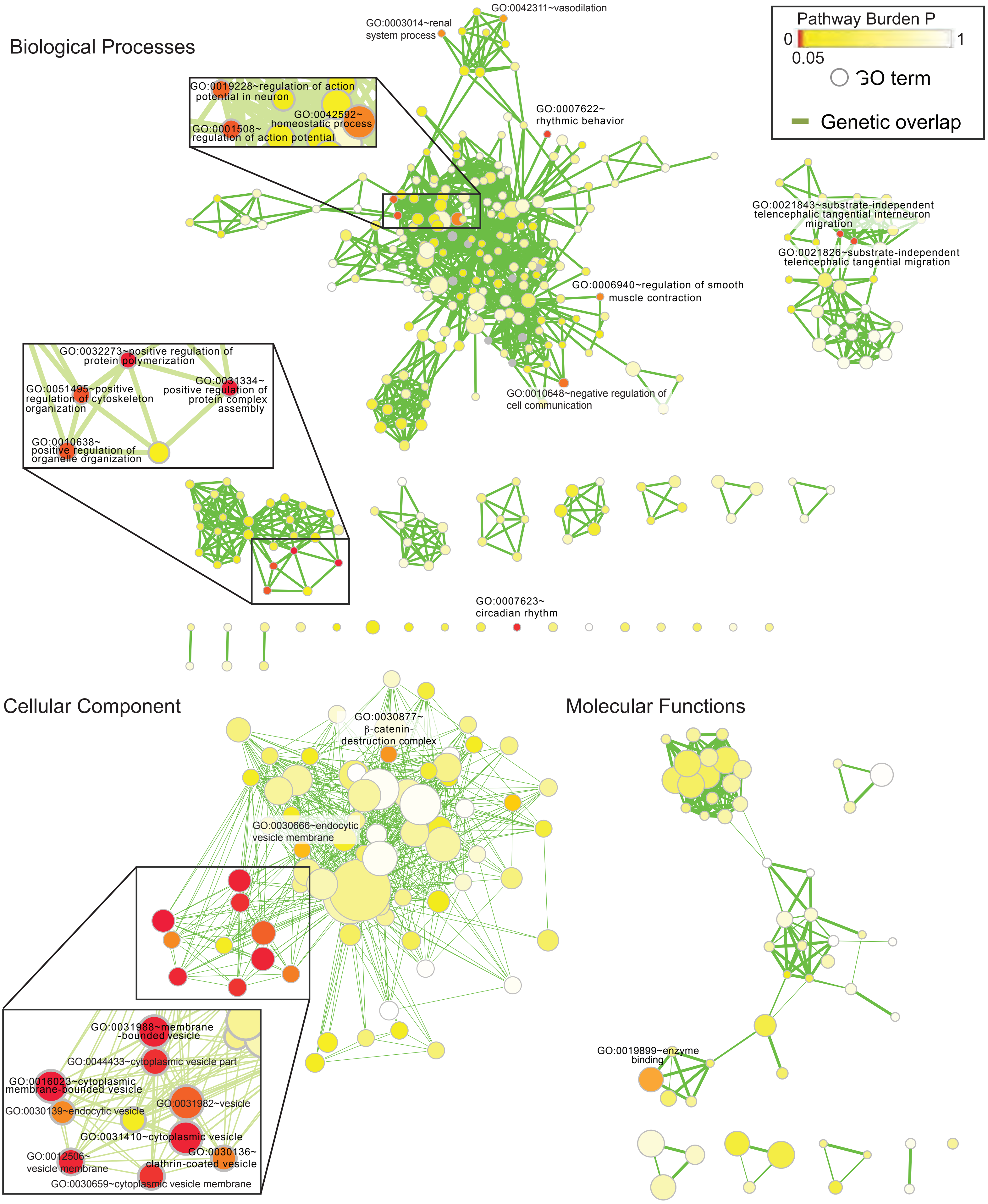

Supplementary Figure 6g. Newtork visualization of the 989 GO sets overlaid with pathway burden P-values

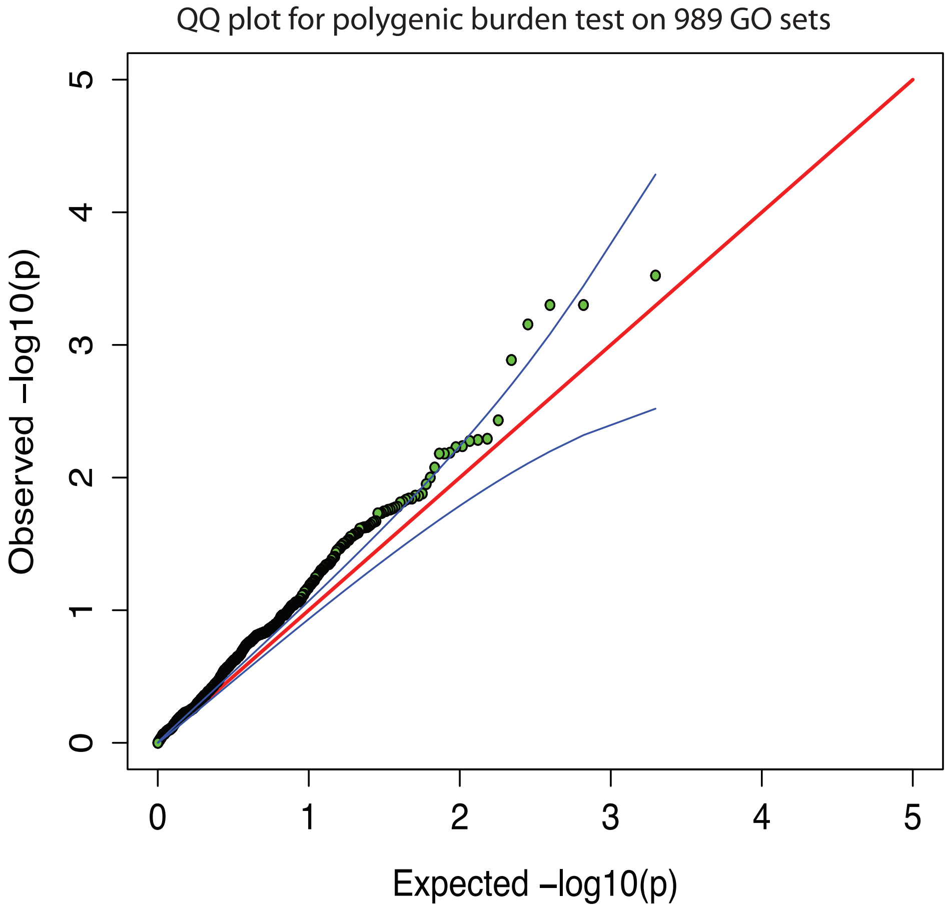

Supplementary Figure 6h. Quantile-quantile plot for pathway-based burden test. Y-axis indicates observed burden p-values. X-axis indicates theoretical p-values expected by chance

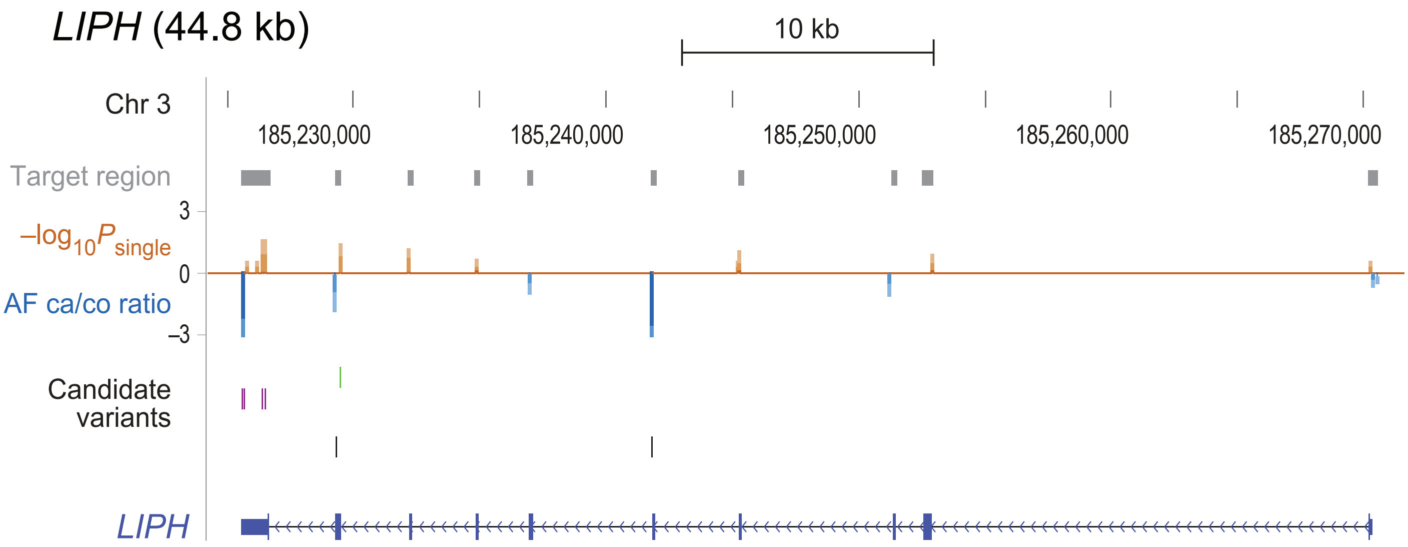

Supplementary Figure 7a. Candidate variants in LIPH

3 coding/1nonDHS cluster

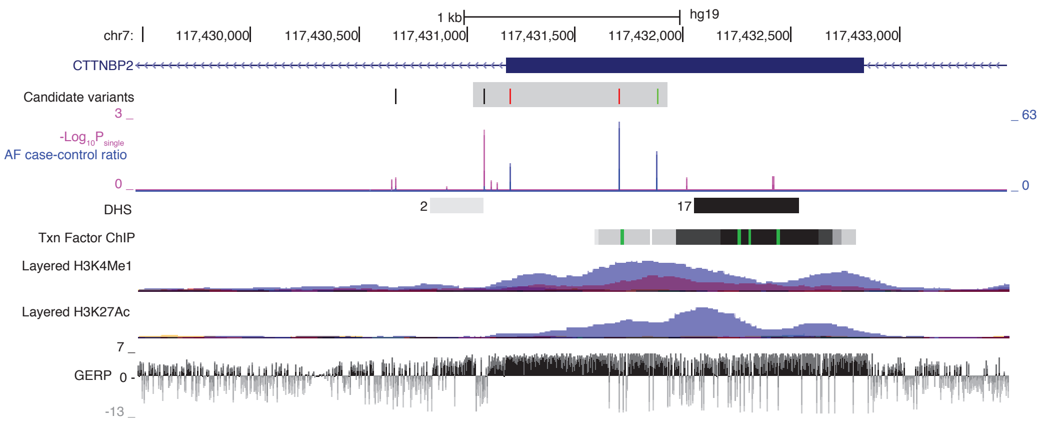

rs12706157/rs13242822

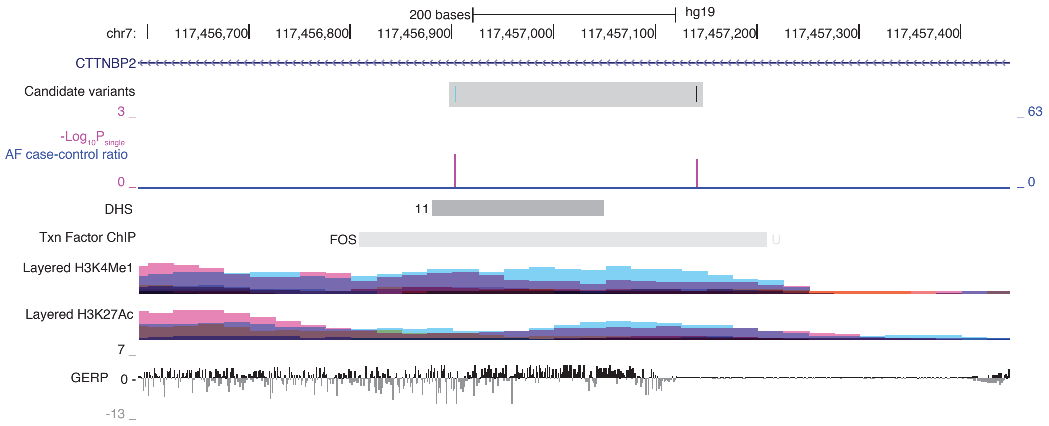

rs2067080/rs2111209

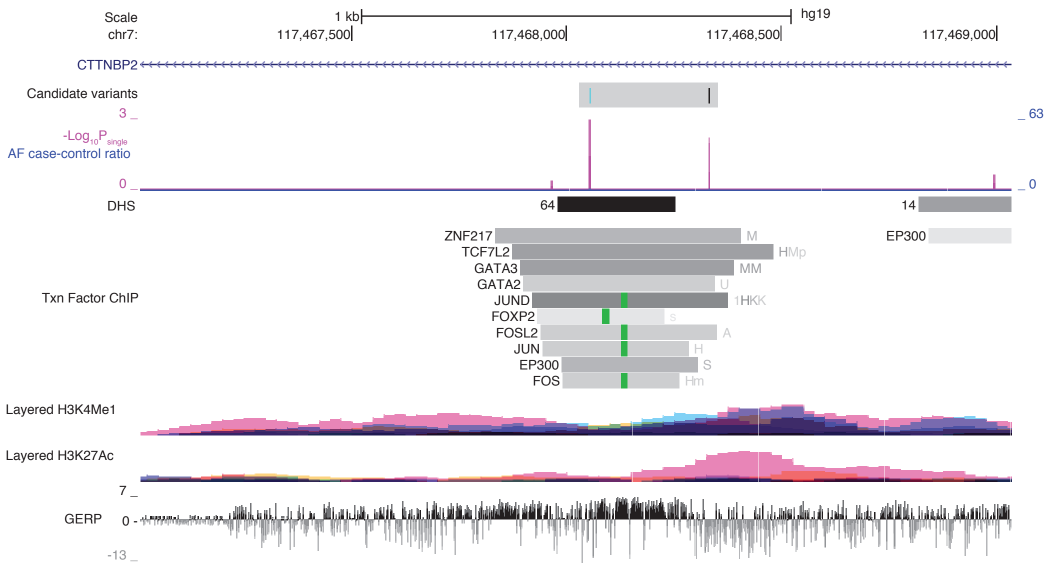

Supplementary Figure 7b. Additional regulatory candidate variants in CTTNBP2

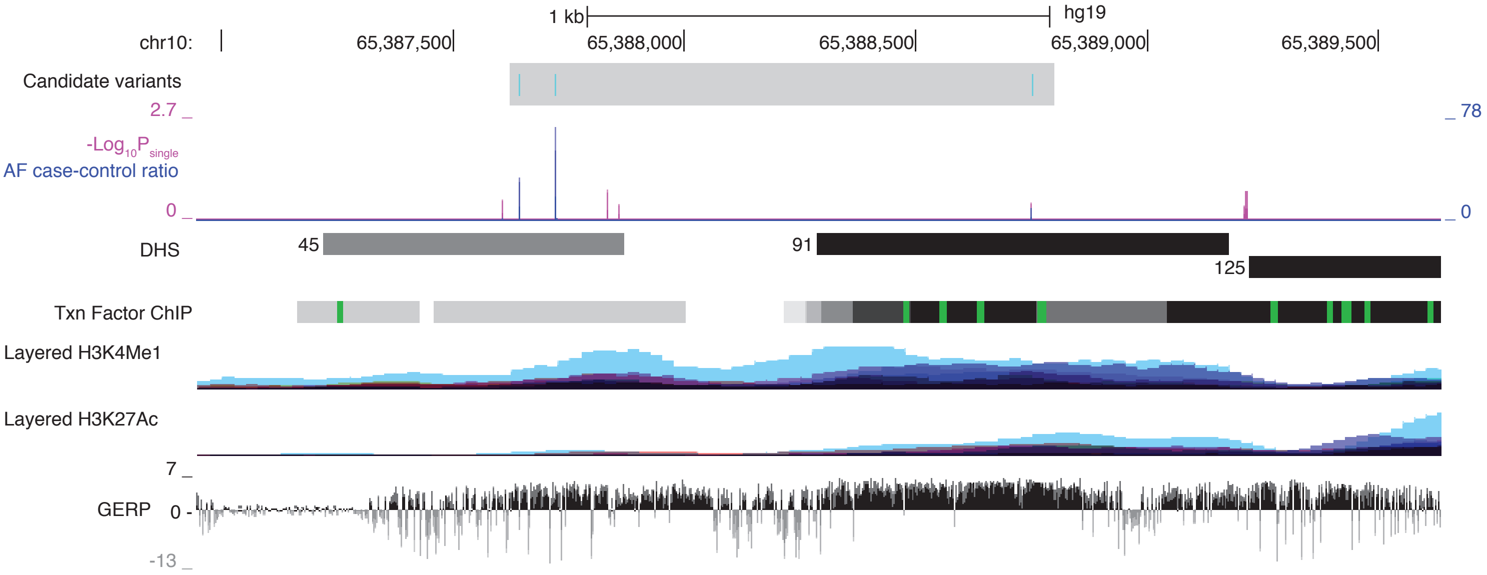

Supplementary Figure 7c. 3kb upstream regulatory candidate variants in REEP3

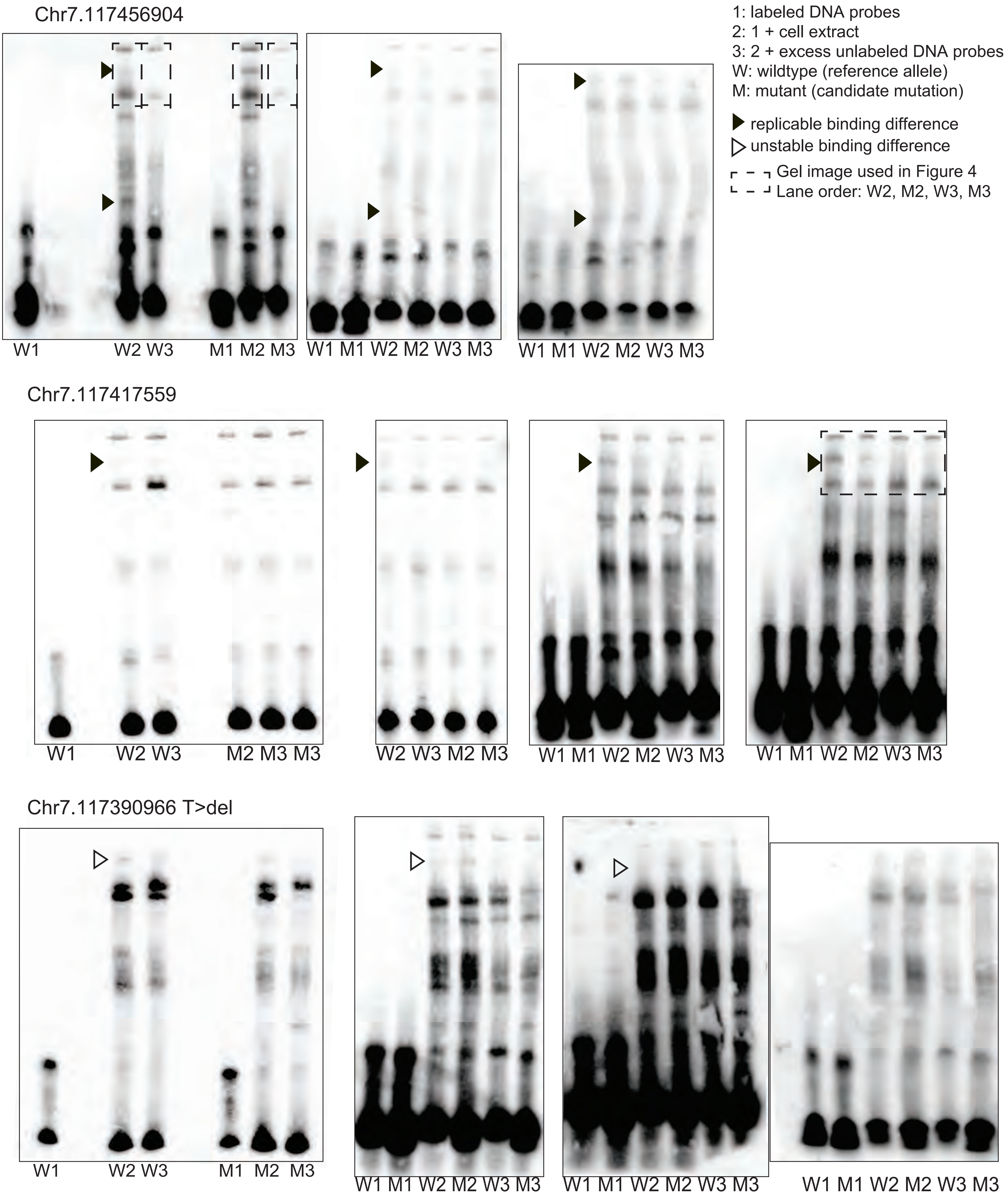

Supplementary Figure 8 . EMSA raw images and replicates showing weak/clear evidence of transcription factor-DNA binding changes by candidate regulatory variants

Continued from previous page.

Chr7.117356081

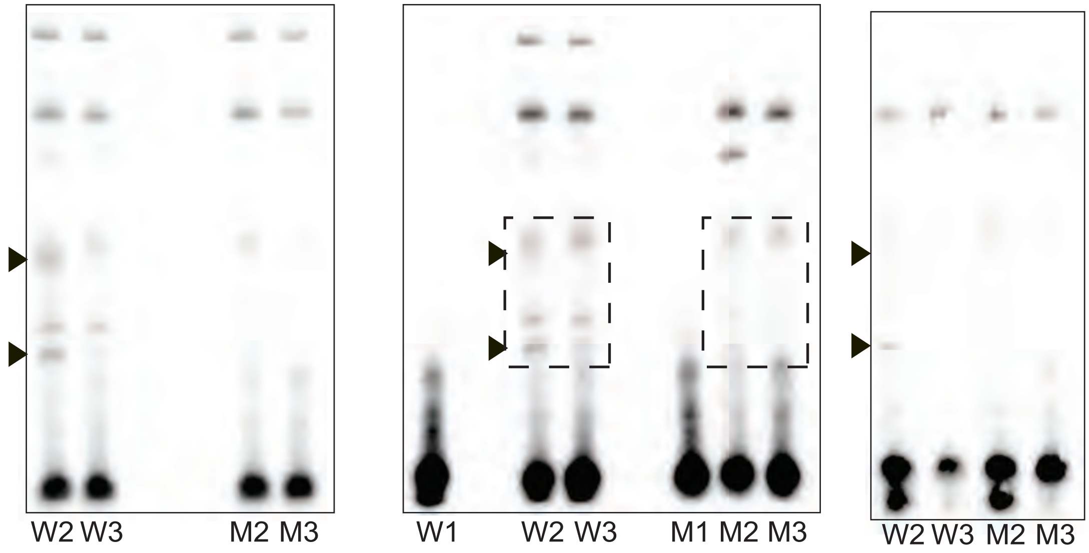

Chr7.117421141 C>A

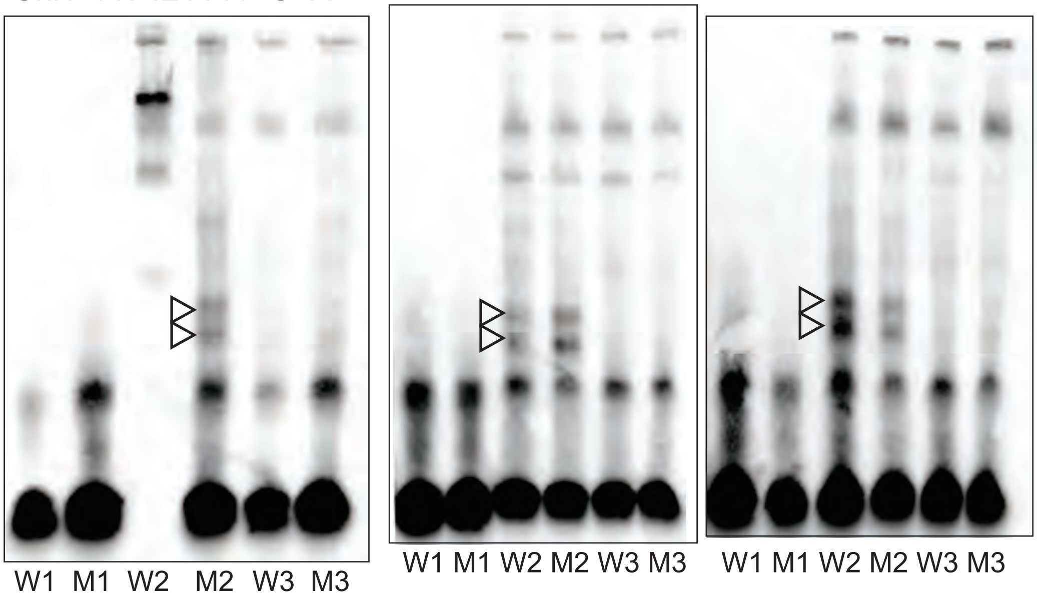

Chr10.65307923

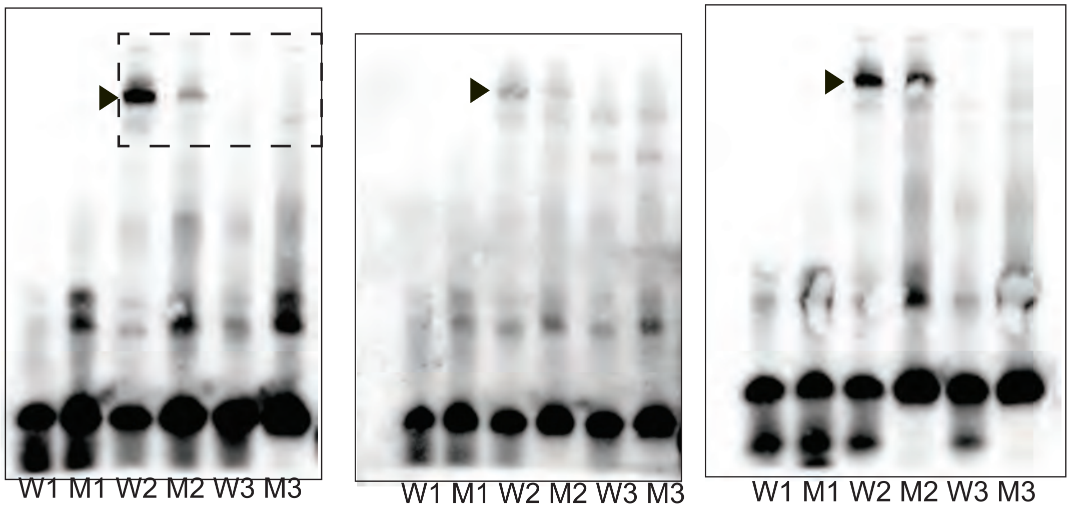

Continued from previous page.

Chr10.65332906

W4: W3 + unlabeled MT DNA probes

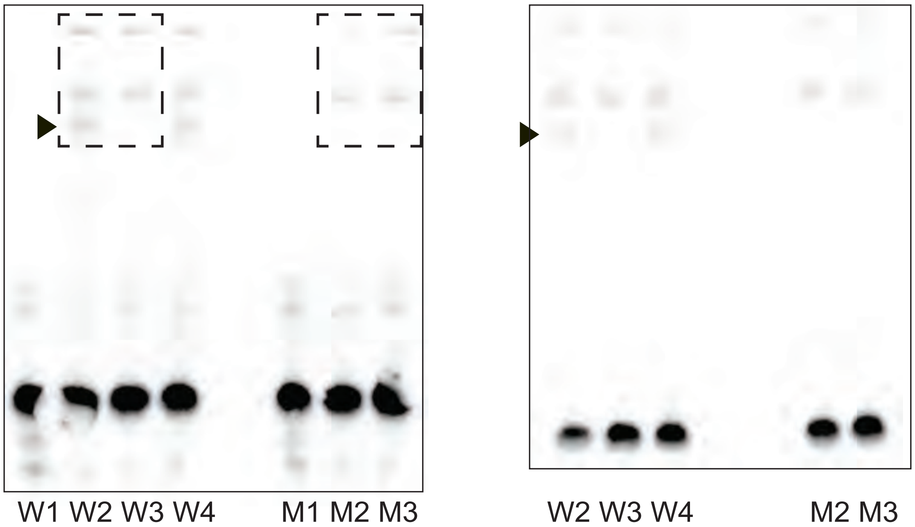

Chr7.117431704 C>T

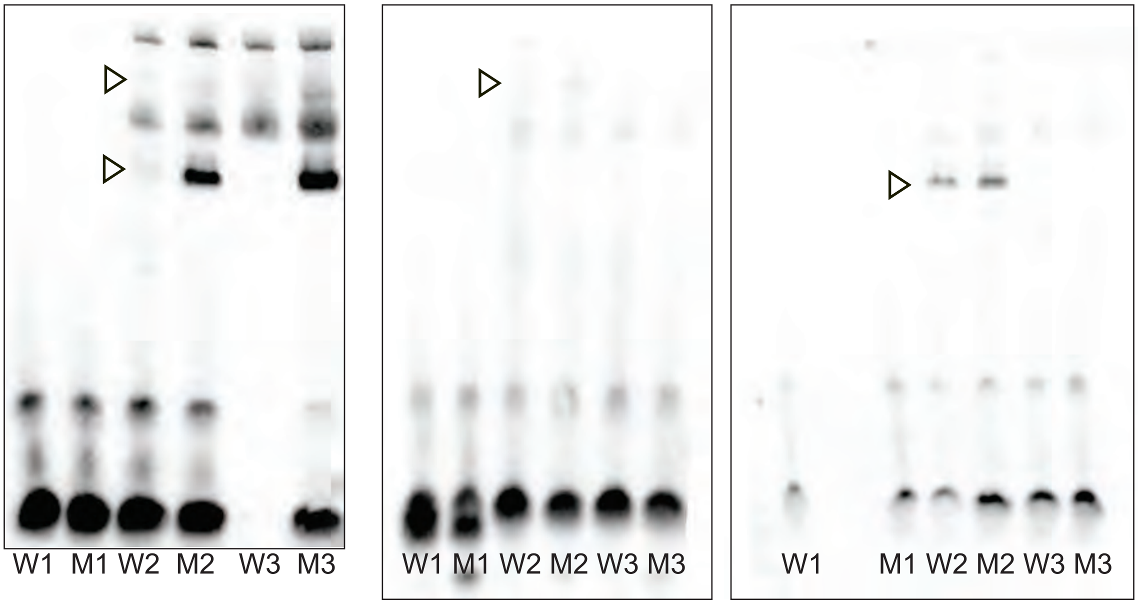

Chr7.117431879

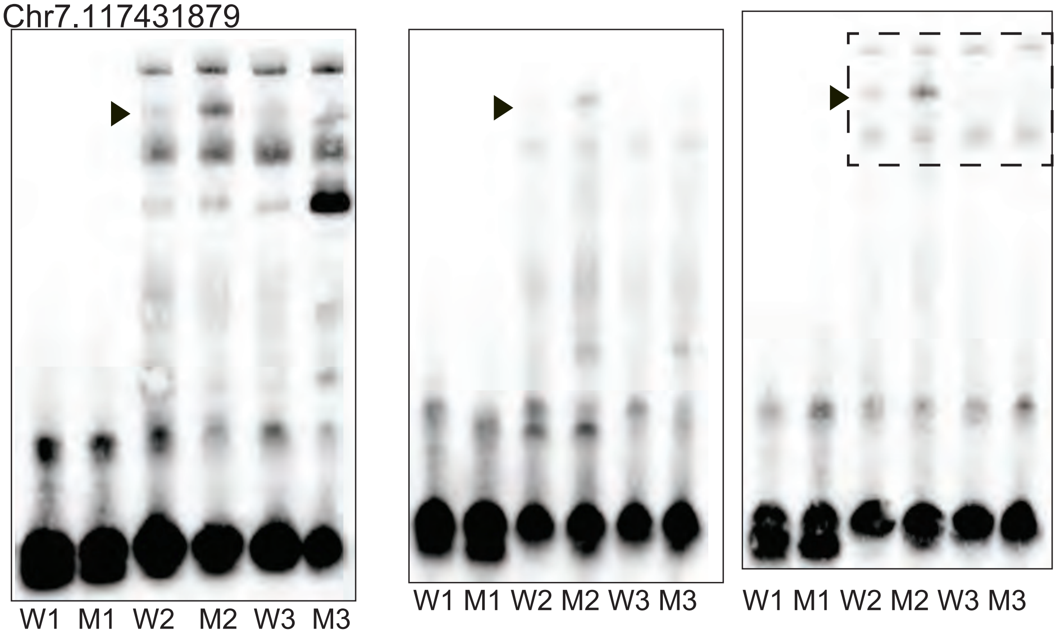

Continued from previous page.

Chr10.65387722 C>del

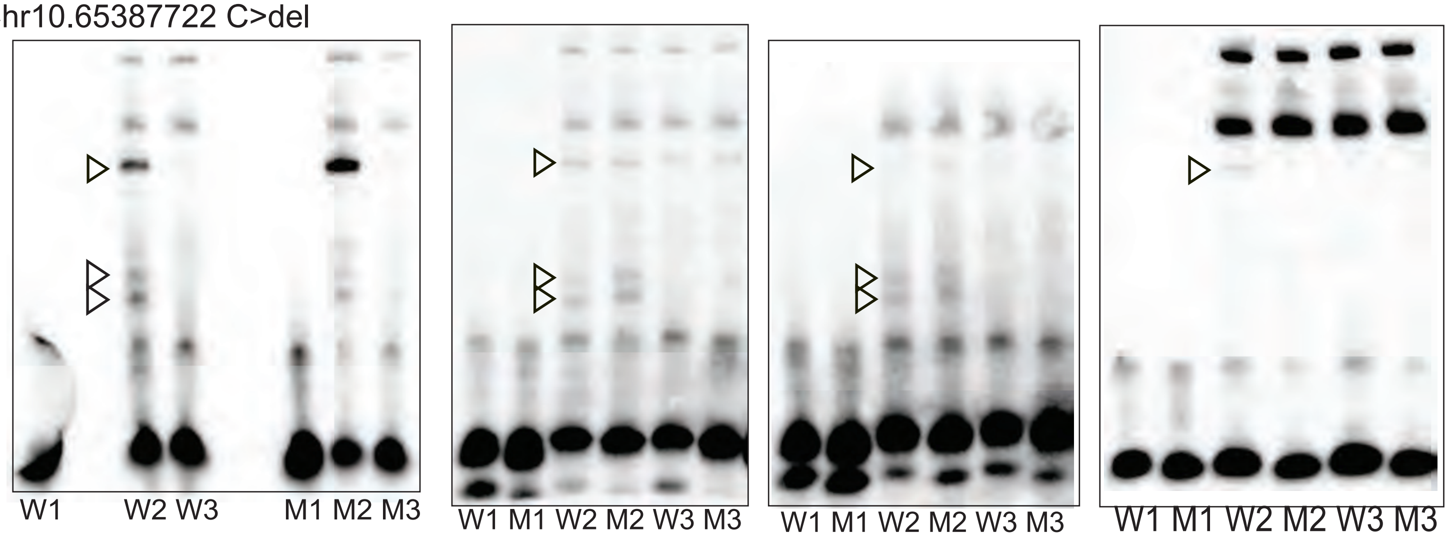

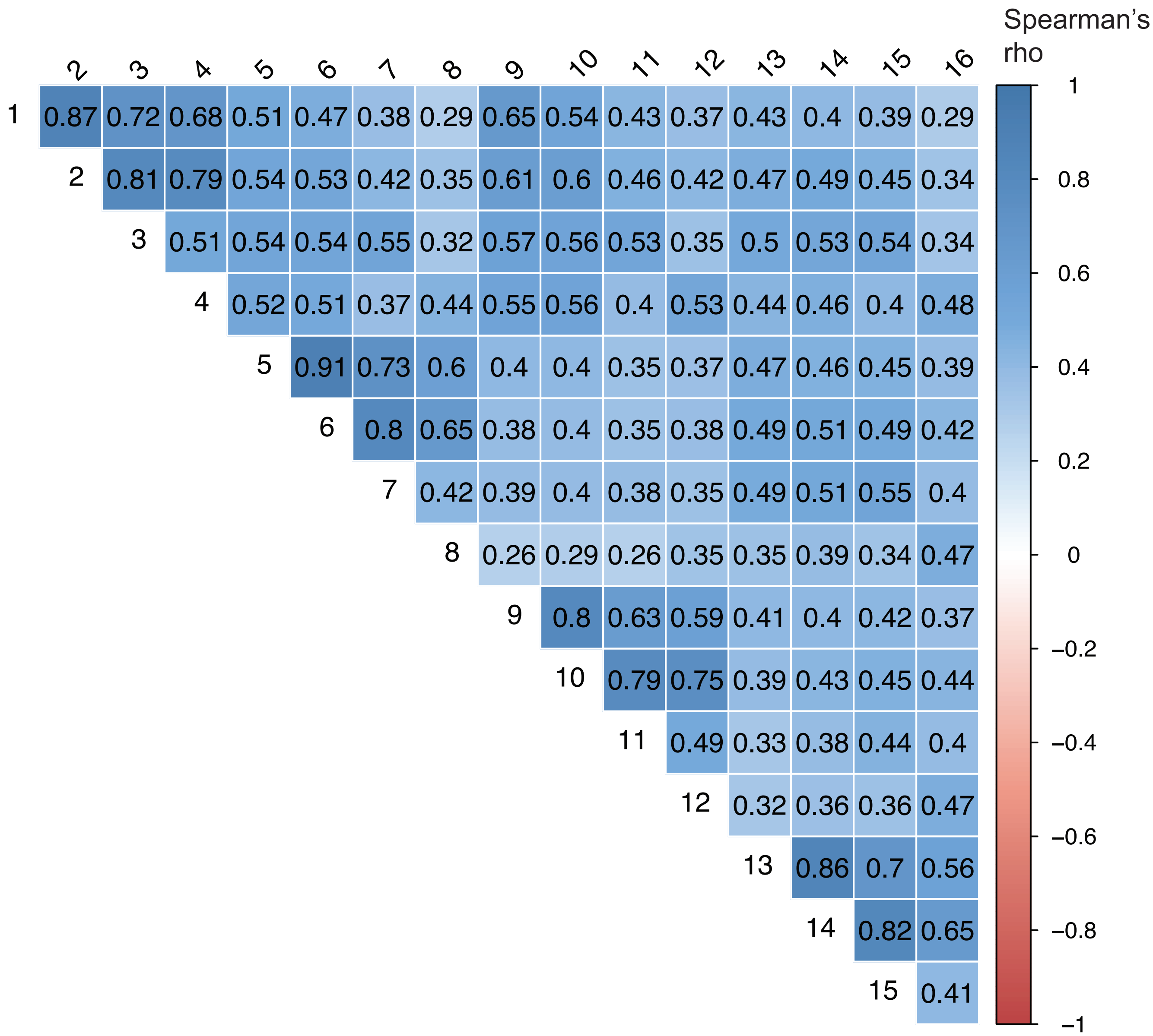

Supplementary Figure 9. Correlation between gene-based burden tests
1-4. Overall-All, -Evolutionary, -Conserved, and -Divergent;
5-8. Exon-All, -Evolutionary, -Conserved, and -Divergent;
9-12. DHS-All, -Evolutionary, -Conserved, and -Divergent;
13-16. Rare-All, -Evolutionary, -Conserved, and -Divergent

SUPPLEMENTARY METHODS

Selection of targeted genes

We compiled a list of 608 candidate genes for OCD (Table S1), based on genetic and neurobiological evidence from dog, mouse, and human studies. 13% (78) of the genes were included based on findings from more than one source.

Our list includes:
(a) 68 genes that encode proteins differentially expressed between dopamine receptor type 1 expressing medium spiny neurons (D1R+MSNs) and dopamine receptor type 2 expressing (D2R+) MSNs (preferentially expressed in D1R+MSNs)[1], based on findings that the imbalance of the activity between direct pathway (composed of D1R+MSNs) and indirect pathway (composed of D2R+MSNs) of the CSTC circuitry may be responsible for OCD[2]. According to the CSTC-OCD model, constructed based on extensive functional neuroimaging and anatomical evidences in humans[3,4], a shift of balance favoring activity in the direct pathway will "disinhibit" the thalamus and thereby promote the selection of behavioral sequences, whereas a shift favoring the indirect pathway has a net effect of reinforcing the inhibitory tone to the thalamus, thereby inhibits the selection of behavioral sequences. Therefore, persistent activation of the direct pathway may lead to inappropriate, potentially repetitive release of cognitive and motor sequences.

(b) 154 genes that encode striatum-enriched postsynaptic density proteins[5–7], based on findings that both *Sapap3* and *Slitrk5,* the genes that exerted OCD-like behaviors in mice when disrupted, encode postsynaptic density proteins that are highly expressed in the striatum[8,9]. Furthermore, selective expression of *Sapap3* in the striatum rescued the compulsive overgrooming and cortical-striatal synaptic defects of the *Sapap3* null mice[8].

(c) 56 human genes whose canine orthologs are located in canine CD GWAS loci[10]. The associated loci were identified as previously published[10]. Briefly, regions were defined using linkage disequilibrium-based clumping around SNPs with $P<0.0001$ (that is, SNPs within 1Mb with $r^2>0.8$ and $P<0.01$). These thresholds were chosen because the canine GWAS was performed within a single dog breed (Doberman pinscher), and dog breeds have extensive linkage disequilibrium and large haplotype blocks (~500kb-1Mb)[11].

(d) 196 autism spectrum disorder (ASD) genes that were available from SFARI Gene (https://gene.sfari.org) as of 2009. We including ASD genes because of the overlapping repetitive behavioral component in OCD and ASD, as well as the high rates of comorbidity (OCD diagnosed in 30-40% of ASD patients)[12,13].

(e) 56 OCD candidate genes from a Pubmed search with keyword 'OCD association' as of 2009.

(f) 91 genes were included from an OCD linkage study[14].

(g) 69 genes were included from other neuropsychiatric disorder-associated chromosomal regions, i.e. 22q11.2[15], 16p11.2[16,17], 15q11.2[18,19], and 8p[20].

Sample information and potential confounders

Study population: Our discovery sample cohort for targeted sequencing consisted of 592 OCD cases of European ancestry by self-report and 560 control individuals of the same ethnicity. The sex ratio (F:M) was 1.05 for cases and 1.1 for controls (data available for 94% of the samples) and the age of ascertainment distribution (Min:Mid:Max) was 11:36:69 for cases and 17:42:92 for controls (data available for 60% of the samples). Each case was evaluated by expert clinicians to confirm an OCD diagnosis using the Structured Clinical Interview for DSM-IV[21], supplemented with the checklist and scores from the Yale-Brown Obsessive Compulsive Scale (Y-BOCS)[22].

Among the 1,152 individuals in the discovery cohort, 597 had been confirmed for their genetic European ancestry in the IOCDF GWAS using the whole genome data[23]. Multidimensional scaling (MDS) analysis of 40 ancestry-informative markers (AIMs) on the 555 non-GWASed individuals, together with HapMap3 populations, resulted in three separable clusters, i.e. European/European admixture, Asian, and African (by J. Chaponis). The analysis showed that only seven individuals fall into Asian or African clusters, with a similar number of cases and controls in each cluster. Given their self-reported ancestry and the variability in genetic ancestry estimates by a small

number of AIMs[24], these individuals, who are not clearly separable from European cluster, are more likely than not to be of European ancestry.

An additional 729 DSM-IV/Y-BOCS OCD cases of Northwestern European ancestry (apparent self-reporting Dutch, Swedish, Swiss, German, or European American; see table below) and 1,105 controls of European ancestry were included as validation samples. The sex ratio (F:M) was 1.01 for cases and 1.08 for controls (data available for 99.8% of the samples), and the age of ascertainment distribution (Min:Mid:Max) was 5:25:79 for cases and 12:43:93 for controls (data available for 70% of the samples). The full set included 1,321 cases and 1,665 controls with sex ratio (F:M) of 1.02 for cases and 1.09 for controls (data available for 98% of the samples) and age of ascertainment distribution (Min:Mid:Max) of 5:27:79 for cases and 12:43:93 for controls (data available for 66%). Informed consent was obtained from all subjects included in our study.

Number of samples contributed by investigator / location

Phase	Investigator (location)	OCD	Control
Discovery	MW/HJG/RS (Bonn, Germany)*[25]	192	196
Discovery	JMS/SES/MJ (Boston, MA, USA),<br>SR (Providence, RI, USA),<br>CAM (San Francisco, CA, USA), and<br>CNP/MTP/JAK (Los Angeles, CA, USA)[23]	400	364
Validation	MW/HJG/RS (Bonn, Germany)*[25]	8	4
Validation	CR (Stockholm, Sweden)*[26]	116	0
Validation	CAM (San Francisco, CA, USA)	64	29
Validation	CNP/MTP/JAK (Los Angeles, CA, USA)*	0	605
Validation	DC (Amsterdam, Netherlands)<br>– NOCDA cohort[27]	266	36
Validation	GLH (Ann Arbor, MI, USA)*[28]	66	0
Validation	CH (Stockholm, Sweden)	0	400
Validation	SW/EG (Zurich, Switzerland)*[29]	209	31
Total		1321	1665

*Confirmed sites that excluded comorbidities of other major psychiatric/neurological conditions

DNA pooling and potential confounders: 16 phenotype-matched individuals were pooled together to create 37 OCD pools and 35 control pools. The number of individuals per pool was determined considering the sequencing error rate (0.5-1% per base) to distinguish singletons from machine errors. The indexed pools were again pooled together and underwent Illumina multiplex sequencing to minimize potential batch effect by different lanes. Reads were aligned and processed by Picard analysis pipeline (http://broadinstitute.github.io/picard/). All pools had at least 95% of the target regions at >30x read depth coverage.

The number of variants detected in each pool was highly comparable across all pools, with >99% of the total detected variants found in all individual pools. Overall, we did not observe a case/control difference in total number of detected variants and in AF distribution. MDS analysis was performed using 1,000 randomly selected variants from the sequence data, and 71/72 pools clustered into one cluster with well-distributed case/control or experimental wave labels.

We also used hierarchical agglomerative clustering and computed significance via multiscale bootstrap resampling implemented in pvclust, in order to form matched groups of pools based on distance (correlation) of AFs. In our data set, two significant clusters were detected from hierarchical agglomerative clustering: one containing the same single outlier case pool detected in the above MDS analysis, and the other containing the remaining 71/72 pools. The outlier pool showed no substantial differences in sequencing quality and read depth compared to the other pools, suggesting that the outlier may be due to a fine-scale sub-population structure. Of note, the outlier pool did not

contain any of the seven outlier individuals from the MDS analysis based on sparse AIMs data. Given that hundreds of random SNPs provide better estimates of genome ancestry than a few dozens of AIMs[24], European ancestry for these individuals thus cannot be completely ruled out.

To exclude the possibility of spurious associations driven by this single outlier pool, we performed gene-based burden tests both including and excluding the outlier pool, and the significantly associated genes did not differ: the same five genes achieved significant associations after multiple testing corrections, with the same variant type enrichments for each associated gene (corr. p<0.053, uncorr. p<0.0008; see table below).

Variant burden of five genes excluding outlier individuals

Genes	PolyStrat in sequencing excl. outlier pool
LIPH	Overall (6×10^{-4})
NRXN1	Exon-Cons (2×10^{-4})<br>Exon-All (4×10^{-4})<br>Exon-Evo (1×10^{-4})
HTR2A	Exon-Cons (7×10^{-4})
CTTNBP2	Overall (7×10^{-4})<br>DHS-All (9×10^{-4})
REEP3	Overall-Div (3×10^{-4})<br>DHS-All (3×10^{-4})

In conclusion, we found no discernable population structure between cases and controls that would explain our significant gene associations. Allele frequencies in both case and control pools were nearly identical to 33,370 non-Finnish Europeans in ExAC (7358 SNPs, Pearson's rho=0.995, $p<2.2\times10^{-308}$). Principal component analysis of individual genotype data for 40 ancestry-informative markers found just 7 of 1152 individuals (0.6%) with potential non-European ancestry in the pooled sequencing cohort. Finally, clustering the pooled sequencing data using 1000 randomly selected SNPs, both rare and common, revealed just one potential outlier pool. To confirm that low-level ancestry differences did not affect our results, we reran PolyStrat excluding this pool and found the same five significantly associated genes.

While we tried to minimize any potential confounders by well-matched case/control selection and careful sequencing experiment design, as well as clustering analysis, due to the limitations arising in the nature of targeted, pooled sequencing strategy, a hidden fine-scale sub-population structure or an inclusion of individuals of a different ancestry on our sequence data could not be fully scrutinized at the individual sample level. Therefore, the follow-up genotyping was particularly useful to minimize possible spurious associations.

Gene-based burden analysis

Variant annotation: We used ANNOVAR[30] to annotate variants (-buildver hg19) and to download relevant datasets, if available in the ANNOVAR database. Coding, synonymous, and non-synonymous status were annotated based on RefSeq genes using options --geneanno -dbtype refGene. Conserved sites (GERP++>2) were annotated using options --filter -dbtype gerp++gt2. Divergent sites (GERP++<-2) in the targeted regions were downloaded from the UCSC Table Browser and annotated using options --regionanno -dbtype bed. DHS sites were annotated using options --regionanno -dbtype wgEncodeRegDnaseClustered, and population frequency and variant novelty from 1KG data were annotated using --filter -dbtype 1000g2012apr all. Annotation of candidate regulatory variants with Roadmap Epigenomics data was done using RegulomeDB 1.1[31]. The overlaps between candidate variants and epigenetic marks and evolutionary sites were visualized using UCSC genome browser's ENCODE and conservation tracks and WashU EpiGenome Browser's Roadmap Epigenomics tracks.

Burden test: To evaluate gene-based burden of variants in cases, we employed a published approach[32]. We used the sum of the differences of non-reference allele rates between cases and controls per gene as test statistic, and calculated the p-values by comparing with the data generated by 10,000 permutations of case-control labels. The test

was performed in 1-sided manner, expecting a greater burden of non-reference alleles in cases than in controls.

Null distribution: We calculated the null distribution of p-values using an empirical model for gene-based burden tests and a theoretical model for pathway-based burden tests. For the theoretical null, uniform distribution was assumed. For the empirical null, we generated test statistics from 100 iterations of the tests identical to the actual association tests on case-control permuted data. We then compared the expected values with the observed values on a quantile-quantile plot and evaluated how much our observed data deviate from the expectation globally.

Multiple testing correction: PolyStrat p-values are corrected for multiple testing using a published permutation-based method that computes empirical experiment-wide significance threshold[32,33]. This multiple testing correction accurately measures statistical significance across correlated gene-based tests, while controlling for type 1 errors. To create an empirical null distribution, we calculated possible minimal p-values for all 9,728 tests (608 genes ✕ 16 categories), and considered as significant real data residing in the top 5% of the null distribution. For most variant categories, quantile-quantile plots revealed good correspondence between observed values and the empirical null, with a small number of genes exceeding the expected distribution in a subset of the burden tests (Supplementary Figures 3b-4). Our empirical correction method produces a meaningful test statistic[33] and is preferable to Bonferroni correction, which assumes that each test is independent. Because variants overlap between the categories, our tests are not independent. Furthermore, the effective number of tests is further reduced because the burden test requires sufficient variants to achieve the asymptotic properties for the test statistic[33].

Specifically, we employed the empirical 'minP' procedure[32,33] to control for multiple testing in gene-based and pathway-based burden tests. For gene-based tests, we jointly corrected for all 16 filters, (i.e. 'Overall', 'Exon', 'DHS' and 'Rare' categories and their four sub-categories stratified by evolutionary status) and for all 608 sequenced genes. The empirical null was generated from the minimum significance ('minP') obtained from case-control permuted datasets for all 16 filters, as if the permuted dataset was the observed dataset. We then evaluated whether the observed p-values fall within the top 5% of 'minP', and the observed p-values that are within top 5% were considered to pass significance threshold. For pathway-based tests, we corrected for all 989 tested GO sets, using the same procedure.

Gene length bias: Gene length can be a confounding factor when counting the number of SNPs, as, theoretically, longer genes tend to carry more SNPs. The permutation approach described in the main text controls successfully for the potential bias, since the gene length and the distribution of the resulting p-values reveal no correlation in our data set (Figure below). Specifically, a linear regression of the gene-based p-values and the associated gene lengths results in a slope that is close to zero (slope = -5.54×10^{-10}, intercept=0.7).

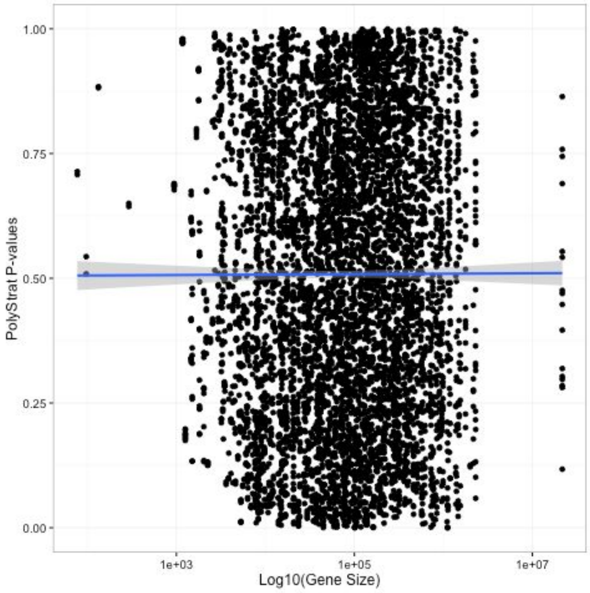

Supplementary Figure 3a. Linear regression analysis of gene-based p-values and gene size.

Variant validation by Sequenom genotyping: Of the 37 SNPs with high quality Sequenome genotype data from the individuals in our discovery cohort, the direction of AF differences between OCD and control were confirmed for all but five variants (Table S1). This is because two variants (chr2:50463984G>A, chr3:185270290C>G) were found to have an extra copy of the non-reference allele in genotyping data (one in case and one in control), and three (chr7:117400406G>T, chr7:117449206C>A, and chr10:65297369T>G) had missing genotypes that were markedly skewed towards cases, with 3-4 times more missing cases than controls (9-18 [control] vs. 36-48 [case]). Of the nine putative doubletons or singletons detected in our pooled sequencing (i.e. estimated sequencing AF<0.0009), only one non-reference allele in control (chr3: 185270290C>G) was not present in the genotyping data, possibly due to missing genotypes or miscalling from the sequence data.

In summary, all SNPs for which the direction of AF difference could be confirmed (32/37), had genotype data shifted in the same direction as in the sequence data. The remaining five variants cannot be confirmed either because genotypes are missing, with skew toward cases, or because an extra copy of the SNP was detected by genotyping.

LD analysis: Gene-based variant burden tests can be confounded by linkage disequilibrium (LD), whereby neighboring variants are inherited together because of population history rather than independent association with the trait of interest. Using the individual genotype data (37 SNPs), we measured LD by calculating the pairwise r^2 for all pairs of SNPs within our five associated genes. Only one pair of SNPs, in the gene LIPH, was strongly linked (defined as $r^2>0.8$). Thus, with the exception of the two SNPs in LIPH, the case-abundant variants independently contributed to the gene burden tests, and significant gene associations in *NRXN1, HTR2A, CTTNBP2* and *REEP3* were not skewed by population structure.

Pathway-based burden analysis

In order to identify gene sets of specific biological relevance for OCD, we performed pathway-based burden analysis. Our pathway burden test is different from GO enrichment test in that we directly evaluated a burden of non-reference alleles in cases, instead of enrichment of genes, in a gene set. This allows us to identify specific subset of genes within our target space that are associated with OCD. An equivalent approach has been employed to detect polygenic burden of disruptive variants in schizophrenia[32].

Gene Ontology (GO) sets: We used GO categories to obtain comprehensive gene sets of biological relevance that represent our genetic search space. DAVID 6.7[34] was used to compute the enrichment of our targeted 608 genes for all GO sets. 989 GO sets that showed weak enrichment (nominal p<0.1) were generously deemed to represent our targeted genes, thus selected for testing pathway associations with OCD.

To study the background functions of the 989 GO sets, we used a treemapping method provided by REVIGO[35] with default parameters (*i.e.* allowed similarity, Medium [0.7]; GO term weight, enrichment p-value; database with GO term size, whole UniProt; semantic similarity measure, SimRel; treemap option, abs log10 p-value), which clustered GO sets based on similarity. This analysis showed that the 989 GO sets cover a range of brain-related functions, from synaptic transmission and ion channel activity to glutamate and dopamine signaling, as well as non-brain-specific terms such as regulation of metabolic processes and cytoskeleton organization (Figure S6a-c).

In order to understand the relationships between these GO sets, we employed a network generation algorithm that is optimally designed for visualizing many highly-related gene sets[36], using CytoScape 3.1.0[37] Enrichment Map Plugin. Nodes and edges were automatically placed based on the parameters recommended by the Enrichment Map manual, *i.e.* enrichment p-value cutoff for building enrichment map, 0.05; overlap metric, Jaccard coefficient; Jaccard coefficient cutoff for building maps, 0.25. The network was arranged by force directed layout weighted mode, using only the interactions that passed the threshold for the similarity coefficient. This automatically placed 415 less-redundant GO sets as nodes and created 1,942 connecting edges, determined by genetic overlaps between two GO sets (Figure S6g).

Burden test:We performed burden tests on the 989 GO gene sets, using the same method as the gene-based burden tests, but evaluating the burden of variants at pathway level, instead of gene level. The overall test results were

moderately inflated compared to the theoretical null, possibly due to the functional grouping of genes (GO sets) that are relevant to OCD (Figure S6h). The top five GO sets, which deviate even more from the null, include three GO sets related to regulation of cell death (GO:0010942, GO:0043065, and GO:0043068, p=3×10^{-4}~5×10^{-4}, corr. p<0.03~0.05), positive regulation of protein complex assembly (GO:0031334, p=7×10^{-4}, corr. p<0.06), and anatomical structure homeostasis (GO:006024, p=1.3×10^{-3}, corr. p<0.1). Additional functional themes from the 82 GO sets with nominal burden (p<0.05), include endocytosis, rhythmic process, cytoplasmic membrane-bounded vesicle, tight junction, and protein kinase binding (Figure S6d-f). Additionally, overlaying the pathway burden test results onto the GO term network topology allowed us to identify clusters of GO sets with strong p-values, such as regulation of protein polymerization and cytoskeleton organization, regulation of action potential, telencephalic tangential migration, and membrane-bounded vesicle (Figure S6g).

Genotyping assay
To optimize Sequenom assay, 46 variants that resulted in significant PolyStrat results and 218 candidate variants were ranked by: i) whether the variant contributed to the specific gene-based tests that produced significant PolyStrat results and ii) single variant association-level (two-sided t-test comparing the estimated AFs between 37 case and 35 control pools). The variant ranking was then used to prioritize variants for designing pools of Sequenom assays to include a similar proportion of top variants for each gene, while maximizing the total number of variants being assayed. Three Sequenom pools capable of assaying a total of 86 variants were designed. Individuals and variants with high rates of missing data (0.63 per individual, 0.21 per variant) were excluded before association analysis, using PLINK1.9[38]. To detect potential LD in genotyping data, we calculated pairwise D-prime and r^2 values using Haploview with standard parameters (pairwise comparison<500kb, minor AF>0.001, genotype rate>0.75, HW p-value>0.001).

Candidate variant analysis
<u>Candidate variant criteria:</u> To identify likely functional candidate variants of five genes, we first excluded 408 SNPs where the frequency of the non-reference, putative risk allele was higher in the controls. From the remainder, we kept SNPs that met any of the following 'stringent' criteria: i) single variant association p<0.05; ii) observed only in OCD cases; or iii) case frequency >2-fold higher than control frequency. We also retained SNPs that met at least two of the following 'relaxed' criteria: i) single variant p<0.1; ii) case frequency >1.5-fold higher than control frequency; iii) fewer than 2 observations in controls; and iv) novel (not found by the 1000 Genomes Project[39]). In total, 218 SNPs (22.3%) met our criteria for candidate variants (7 in *LIPH*, 152 in *NRXN1*, 16 in *HTR2A*, 33 in *CTTNBP2* and 10 in *REEP3*; Figure 2, Supplementary Figure 7a). We ranked these SNPs by strength of association with OCD and selected the top 30% in each gene for further validation. This totaled 67 SNPs, including 42 rare SNPs (AF<0.01) (Figure 3a).

<u>Gene-based analysis:</u> The table below shows the candidate variant enrichments of five genes in OCD from the genotyping data of the 1st (a subset of 571 cases and 555 controls [98%] of the discovery sequencing samples), 2nd (an independent set of 727 cases and 1,105 controls), and the combined (1st + 2nd) cohort. The "Candidate risk SNPs genotyped" column shows the number of candidate risk variants genotyped for each gene, the middle four columns show enrichment results in different cohorts, and the "Validated candidate risk SNPs (full)" column shows the number of candidate risk variants that are more common in cases than in controls in the combined set. A total of 63 candidate risk SNPs were genotyped, and the enrichment was calculated for each gene by comparing the case AFs and the control AFs, expecting higher AFs in cases, using paired 1-sided Wilcoxon test. Note that the test statistics, the variants included, as well as the samples used to compute variant enrichment in the discovery genotyping data differ from PolyStrat analysis of the discovery sequencing data, explaining the differences between p-values from PolyStrat and genotyping analysis. Given the genetic heterogeneity of the disease, variant enrichments in a 2nd set are expected to be weaker than in a 1st set, as observed in our data.

While *NRXN1*'s variant enrichment was nominally significant in the 2nd set with p=0.019 (based on the comparison of 36 AF pairs), with much fewer AF pairs in the other four genes (4-12 AF pairs), the test may have insufficient power to detect enrichments. As the test compares the distribution of case and control AF pairs, power of the test is heavily influenced by the number of variant sites tested, not by the number of individuals included. Due to such a property, a gene's p-value from the combined set does not necessarily behave as a function of its 1st and 2nd set

p-values.

As an extra caution on potential population structure, we performed the variant enrichment tests on our combined genotyping data, excluding the seven outliers from AIMs analysis, which removed three variants specific to these individuals from the analysis. The exclusion modestly changed the genes' variant enrichment levels, with no p-value changes in *LIPH* and *REEP3*, decrease in *CTTNBP2* (original p=0.003 changed to 0.001) and increase in *HTR2A* (p=0.156 changed to 0.219) and *NRXN1* ($p=7.29\times10^{-7}$ changed to 2.8×10^{-6}). *NRXN1*'s variant enrichment also remains strong ($p=1.45\times10^{-6}$), when excluding a candidate variant sharing a haplotype (defined by D-prime confidence intervals) with another variant in our genotyping data.

Supplementary Table 1c. Five genes' candidate variant enrichments in genotyping data (paired 1-sided Wilcoxon)

Genes	Candidate risk SNPs genotyped	1st cohort (571 cases + 555 controls)	2nd cohort (727 cases + 1,105 controls)	Combined (1,298 cases + 1,660 controls)	Combined excl. 7 outliers	Validated candidate risk SNPs (full)
LIPH	4	0.063	0.813	0.813	0.813	2
NRXN1	36	2.71×10^{-6}	0.019	7.29×10^{-7}	2.8×10^{-6}	32
HTR2A	6	0.109	0.109	0.156	0.219	4
CTTNBP2	12	0.003	0.207	0.003	0.001	10
REEP3	5	0.031	0.313	0.031	0.031	5
Total	63	4.32×10^{-10}	0.005	1.08×10^{-7}	2.13×10^{-7}	53

Protein sequence analysis: For the protein sequence analysis with regards to the impact of candidate coding variants in *NRXN1* and *HTR2A*, a protein sequence of interest containing a candidate variant and the location of corresponding amino acid residues were obtained by blastx on UniProtKB, then protein domain search by InterPro sequence search[40]. Amino acid modification information was extracted from UniProt. The theoretical protein structure model of NRXN1 was generated using MuPIT[41].

Expression of four genes in the striatum: To check whether our top four genes are expressed in the striatum, we extracted 990 log2 expression values of the 33 probes for the four genes, which are measured from 5 sub-regions of the striatum in 6 human individuals (Allen Brain Atlas's microarray data; http://human.brain-map.org/). The data confirmed that all four genes are abundantly expressed in the human striatum (*NRXN1*, mean expression level[$\log_2$]=7.6 from 4 probes in 30 regions; *REEP3*, mean expression level[$\log_2$]=7.9 from 2 probes in 30 regions; *CTTNBP2*, mean expression level[$\log_2$]=8.3 from 2 probes in 30 regions; *HTR2A*, mean expression level[$\log_2$]=4.4 from 25 probes in 30 regions).

Potential impact of synonymous variants: It has been suggested that synonymous variants identified in brain disorders may affect protein folding by disrupting RNA processing and post-transcriptional regulation, or by altering mRNA degradation[42,43]. All our synonymous candidate variants in *NRXN1* reside at the bases that are unusually fast-evolving (chr2:50,463,984, GERP++ –11.4; chr2:50,464,065 GERP++ –10.8, chr2:50,723,068 GERP++ –11.2) or slow-evolving (chr2:50,733,745, GERP++ 3.41; chr2:50,850,686, GERP++ 3.51), suggesting that these variants may have potential regulatory functions.

ExAC analysis

The public ExAC database only provides allele frequencies for variants; our pooled sequencing data has the same constraint. Thus, permutation based approaches (e.g. case/control label swapping) requiring individual-level genetic data are not possible. Instead, we used our control data to construct a null model. Because our allele frequencies are almost perfectly correlated with ExAC (Pearson's rho=0.995, $p<2.2\times10^{-308}$ for 7358 shared variants; Figure 3b,c), we expect no association between our controls and ExAC. However, Fisher's Exact test gives highly significant association signals even for this "null" comparison, due to the extremely large size of the ExAC cohort.

We instead use an isoform-based test, comparing the distribution of variants across different isoforms of the same gene. By incorporating a within-gene comparison to assess significance, we effectively control inflation in the null case. We first calculate, using ExAC, the number of rare (AF<0.01), non-reference, coding alleles within each

isoform. We then use the χ^2 goodness-of-fit test to compare the distribution of variants across isoforms in our data and ExAC.

Evaluating concordance between our control sequencing data and the ExAC data set: To evaluate the concordance between our control sequencing data and the ExAC data at gene level, we examined the sum of standardized Euclidean distances from a point (E,C) to a line y=x, where E is allele frequency in ExAC data, C is allele frequency in our controls, n is the number of nucleotide polymorphisms in a gene, and $y=x$ is a line that consists of points where E=C (i.e. perfect concordance). Standardizing factor (E+C) was used to upweight the rare allele differences and downweight the common allele differences. The total Euclidean distance for a given gene is then calculated by summing the standardized distances calculated for its constituent variants. The resulting formula used for each gene is:

$$\text{Gene-level distance, d} = \sum_{i=1}^{n} \text{Standardized } d_i = \sum_{i=1}^{n} \sqrt{\frac{\{(C_i - E_i)/(C_i + E_i)\}^2}{2}}$$

Under this formulation, in the case of a perfect match between ExAC and Control data ($C_i = E_i$), Standardized d_i =0; in the case of complete mismatch ($C_i = 0$, $E_i = 1$ or $C_i = 1$, $E_i = 0$), Standardized d_i ~0.71; in the case of moderate mismatch, e.g. C_i=0.003 and E_i=0.001, Standardized d_i ~0.35; in the case of severe mismatch, e.g. C=0.001 and E=0.1, Standardized d_i ~0.63. A gene with 15 moderately mismatched variant positions would have $\sum_{i=1}^{n}$ Standardized d_i ~5.25. We evaluated all 608 genes in our study and found that 98% of the genes have $\sum_{i=1}^{n}$ Standardized d_i < 6 (Figure below).

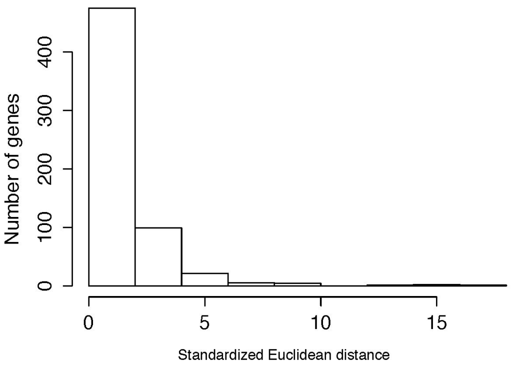

Gene selection for testing: Of our full list of 608 genes, 542 genes had at least two unique ensembl transcripts (Ensembl GRCh37 assembly). The theoretical (i.e. directionality of burden test approach in PolyStrat) and empirical (i.e. distribution of PolyStrat p-values as function of non-reference allele counts per gene) indicated that genes with fewer than three non-reference allele count differences between cases and controls would be incapable of producing association signals by PolyStrat. Thus, for the direct comparison between the original association results with the ExAC analysis regardless the methods used, we restricted the isoform-based ExAC analysis to the 66 genes that are likely to be sensitive to both methods.

Isoform analysis: We examined the residuals of each *NRXN1* isoform to identify the candidate isoform that has the largest difference from the expected values under the ExAC data. We first calculated the raw residuals (Obs-Exp) for both OCD and control data in relative to ExAC data and found that the isoform *NRXN1a-2* (*NRXN1-006*) has the largest residual in our OCD data while having relatively small residual in our control data (Figure below).

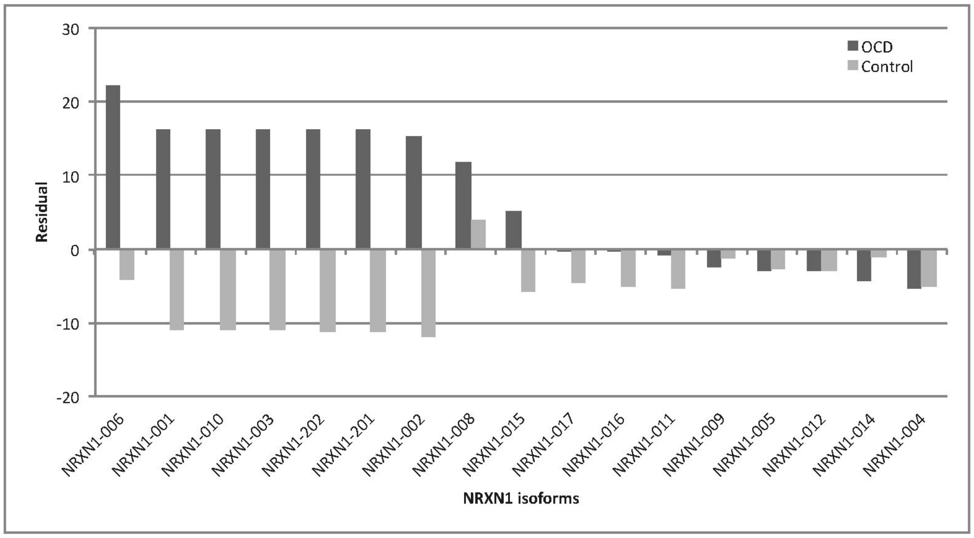

In order to systematically account for the residuals observed in the controls, we calculated the ratio of the residuals (OCD residual/Control residual) for each isoform and ranked the isoforms accordingly (Figure below).

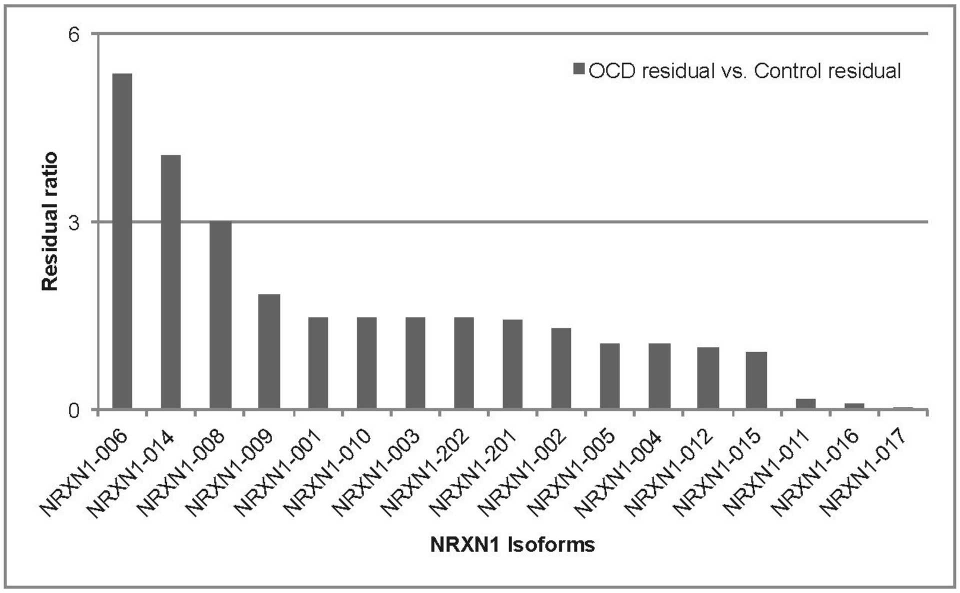

SUPPLEMENTARY REFERENCES

1. Heiman, M. *et al.* A translational profiling approach for the molecular characterization of CNS cell types. *Cell* **135,** 738–748 (2008).
2. Ting, J. T. & Feng, G. Neurobiology of obsessive-compulsive disorder: insights into neural circuitry dysfunction through mouse genetics. *Curr. Opin. Neurobiol.* **21,** 842–848 (2011).
3. Saxena, S. & Rauch, S. L. Functional neuroimaging and the neuroanatomy of obsessive-compulsive disorder. *Psychiatr. Clin. North Am.* **23,** 563–586 (2000).
4. Albin, R. L., Young, A. B. & Penney, J. B. The functional anatomy of disorders of the basal ganglia. *Trends Neurosci.* **18,** 63–64 (1995).
5. Collins, M. O. *et al.* Molecular characterization and comparison of the components and multiprotein complexes in the postsynaptic proteome. *J. Neurochem.* **97 Suppl 1,** 16–23 (2006).
6. Peng, J. *et al.* Semiquantitative proteomic analysis of rat forebrain postsynaptic density fractions by mass spectrometry. *J. Biol. Chem.* **279,** 21003–21011 (2004).
7. Lein, E. S. *et al.* Genome-wide atlas of gene expression in the adult mouse brain. *Nature* **445,** 168–176 (2007).
8. Welch, J. M. *et al.* Cortico-striatal synaptic defects and OCD-like behaviours in Sapap3-mutant mice. *Nature* **448,** 894–900 (2007).
9. Shmelkov, S. V. *et al.* Slitrk5 deficiency impairs corticostriatal circuitry and leads to obsessive-compulsive-like behaviors in mice. *Nat. Med.* **16,** 598–602 (2010).
10. Tang, R. *et al.* Candidate genes and functional noncoding variants identified in a canine model of obsessive-compulsive disorder. *Genome Biol.* **15,** R25 (2014).
11. Lindblad-Toh, K. *et al.* Genome sequence, comparative analysis and haplotype structure of the domestic dog. *Nature* **438,** 803–819 (2005).
12. Leyfer, O. T. *et al.* Comorbid psychiatric disorders in children with autism: interview development and rates of disorders. *J. Autism Dev. Disord.* **36,** 849–861 (2006).
13. Russell, A. J., Mataix-Cols, D., Anson, M. & Murphy, D. G. Obsessions and compulsions in Asperger syndrome and high-functioning autism. *Br. J. Psychiatry* **186,** 525–528 (2005).
14. Shugart, Y. Y. *et al.* Genomewide linkage scan for obsessive-compulsive disorder: evidence for susceptibility

loci on chromosomes 3q, 7p, 1q, 15q, and 6q. *Mol. Psychiatry* **11,** 763–770 (2006).

15. Bassett, A. S., Marshall, C. R., Lionel, A. C., Chow, E. W. & Scherer, S. W. Copy number variations and risk for schizophrenia in 22q11.2 deletion syndrome. *Hum. Mol. Genet.* **17,** 4045–4053 (2008).
16. McCarthy, S. E. *et al.* Microduplications of 16p11.2 are associated with schizophrenia. *Nat. Genet.* **41,** 1223–1227 (2009).
17. Kumar, R. A. *et al.* Recurrent 16p11.2 microdeletions in autism. *Hum. Mol. Genet.* **17,** 628–638 (2008).
18. Stefansson, H. *et al.* Large recurrent microdeletions associated with schizophrenia. *Nature* **455,** 232–236 (2008).
19. van der Zwaag, B. *et al.* A co-segregating microduplication of chromosome 15q11.2 pinpoints two risk genes for autism spectrum disorder. *Am. J. Med. Genet. B Neuropsychiatr. Genet.* **153B,** 960–966 (2010).
20. Tabares-Seisdedos, R. & Rubenstein, J. L. Chromosome 8p as a potential hub for developmental neuropsychiatric disorders: implications for schizophrenia, autism and cancer. *Mol. Psychiatry* **14,** 563–589 (2009).
21. American Psychiatric Association. *Diagnostic and statistical manual of mental disorders : DSM-IV-TR*. (American Psychiatric Association, 2000).
22. Goodman, W. K. *et al.* The Yale-Brown Obsessive Compulsive Scale. I. Development, use, and reliability. *Arch. Gen. Psychiatry* **46,** 1006–1011 (1989).
23. Stewart, S. E. *et al.* Genome-wide association study of obsessive-compulsive disorder. *Mol. Psychiatry* **18,** 788–798 (2013).
24. Pardo-Seco, J., Martinon-Torres, F. & Salas, A. Evaluating the accuracy of AIM panels at quantifying genome ancestry. *BMC Genomics* **15,** 543 (2014).
25. Grabe, H. J. *et al.* Familiality of obsessive-compulsive disorder in nonclinical and clinical subjects. *Am. J. Psychiatry* **163,** 1986–1992 (2006).
26. Andersson, E. *et al.* D-Cycloserine vs Placebo as Adjunct to Cognitive Behavioral Therapy for Obsessive-Compulsive Disorder and Interaction With Antidepressants: A Randomized Clinical Trial. *JAMA Psychiatry* **72,** 659–667 (2015).
27. Schuurmans, J. *et al.* The Netherlands Obsessive Compulsive Disorder Association (NOCDA) study: design and rationale of a longitudinal naturalistic study of the course of OCD and clinical characteristics of the sample at

baseline. *Int. J. Methods Psychiatr. Res.* **21,** 273–285 (2012).

28. Hanna, G. L. *et al.* Evidence for a susceptibility locus on chromosome 10p15 in early-onset obsessive-compulsive disorder. *Biol. Psychiatry* **62,** 856–862 (2007).

29. Walitza, S. *et al.* Trio study and meta-analysis support the association of genetic variation at the serotonin transporter with early-onset obsessive-compulsive disorder. *Neurosci. Lett.* **580,** 100–103 (2014).

30. Wang, K., Li, M. & Hakonarson, H. ANNOVAR: functional annotation of genetic variants from high-throughput sequencing data. *Nucleic Acids Res.* **38,** e164 (2010).

31. Boyle, A. P. *et al.* Annotation of functional variation in personal genomes using RegulomeDB. *Genome Res.* **22,** 1790–1797 (2012).

32. Purcell, S. M. *et al.* A polygenic burden of rare disruptive mutations in schizophrenia. *Nature* **506,** 185–190 (2014).

33. Kiezun, A. *et al.* Exome sequencing and the genetic basis of complex traits. *Nat. Genet.* **44,** 623–630 (2012).

34. Huang da, W., Sherman, B. T. & Lempicki, R. A. Systematic and integrative analysis of large gene lists using DAVID bioinformatics resources. *Nat. Protoc.* **4,** 44–57 (2009).

35. Supek, F., Bosnjak, M., Skunca, N. & Smuc, T. REVIGO summarizes and visualizes long lists of gene ontology terms. *PLoS One* **6,** e21800 (2011).

36. Merico, D. *et al.* Enrichment Map: A Network-Based Method for Gene-Set Enrichment Visualization and Interpretation. *PLoS One* **5,** e13984 (2010).

37. Shannon, P. *et al.* Cytoscape: a software environment for integrated models of biomolecular interaction networks. *Genome Res.* **13,** 2498–2504 (2003).

38. Chang, C. C. *et al.* Second-generation PLINK: rising to the challenge of larger and richer datasets. *Gigascience* **4,** 7 (2015).

39. The 1000 Genomes Project Consortium. A global reference for human genetic variation. *Nature* **526,** 68–74 (2015).

40. Mitchell, A. *et al.* The InterPro protein families database: the classification resource after 15 years. *Nucleic Acids Res.* (2014). doi:10.1093/nar/gku1243

41. Niknafs, N. *et al.* MuPIT interactive: webserver for mapping variant positions to annotated, interactive 3D

structures. *Hum. Genet.* **132,** 1235–1243 (2013).

42. Sauna, Z. E. & Kimchi-Sarfaty, C. Understanding the contribution of synonymous mutations to human disease. *Nat. Rev. Genet.* **12,** 683–691 (2011).

43. Takata, A., Ionita-Laza, I., Gogos, J. A., Xu, B. & Karayiorgou, M. De Novo Synonymous Mutations in Regulatory Elements Contribute to the Genetic Etiology of Autism and Schizophrenia. *Neuron* **89,** 940–947 (2016).
