# Supplementary material for: Integrating evolutionary and regulatory information with a multispecies approach implicates genes and pathways in obsessive-compulsive disorder
Source: Nat Commun. 2017 Oct 17;8:774. doi: 10.1038/s41467-017-00831-x (PMC5645406; doi:10.1038/s41467-017-00831-x)
Supplement: Supplementary file 3 — Supplementary Description [file 41467_2017_831_MOESM3_ESM.pdf]

**Description of Additional Supplementary Files**

File Name: Supplementary Data 1
Description: Gene-based variant burden test results (a) List of 608 Targeted genes. (b) 46 variants used in PolyStrat tests that yielded significant gene-based variant burden. (c) Five genes' candidate variant enrichments in genotyping data.

File Name: Supplementary Data 2
Description: Pathway-based variant burden test results for 989 GO terms.

File Name: Supplementary Data 3
Description: Candidate variants and their frequencies. (a) 218 candidate variants from five genes. Summary genotyping results of these variants in the (b) 1st cohort, (c) 2nd cohort, and (d) combined (1st + 2nd) cohort.

File Name: Supplementary Data 4
Description: Probe sequences for EMSA.
